# Addressing nonlinearities in Monte Carlo

**DOI:** 10.1038/s41598-018-31574-4

**Published:** 2018-09-05

**Authors:** Jérémi Dauchet, Jean-Jacques Bezian, Stéphane Blanco, Cyril Caliot, Julien Charon, Christophe Coustet, Mouna El Hafi, Vincent Eymet, Olivier Farges, Vincent Forest, Richard Fournier, Mathieu Galtier, Jacques Gautrais, Anaïs Khuong, Lionel Pelissier, Benjamin Piaud, Maxime Roger, Guillaume Terrée, Sebastian Weitz

**Affiliations:** 1 Université Clermont Auvergne, CNRS, SIGMA Clermont, Institut Pascal, F-63000 Clermont-Ferrand, France; 2 Université Fédérale de Toulouse Midi-Pyrénées, Mines Albi, UMR CNRS 5302, Centre RAPSODEE, Campus Jarlard, F-81013 Albi CT Cedex, France; 3 LAPLACE, Université de Toulouse, CNRS, INPT, UPS, Toulouse, France; 4 Processes, Materials and Solar Energy Laboratory, PROMES, CNRS, 7 rue du Four Solaire, 66120 Font-Romeu-Odeillo-Via, France; 5 Méso-Star SAS, LONGAGES, France; 6 Université de Lorraine, LEMTA, CNRS, UMR 7563, Vandoeuvre-lès-Nancy, F-54500 France; 7 Univ Lyon, CNRS, INSA-Lyon, Université Claude Bernard Lyon 1, CETHIL UMR5008, F-69621 Villeurbanne, France; 8 Centre de Recherches sur la Cognition Animale, Centre de Biologie Intégrative (CBI), Centre National de la Recherche Scientifique (CNRS), Université Paul Sabatier (UPS), F-31062 Toulouse Cedex 9, France; 9 Education, Formation, Travail, Savoirs (EFTS), Université de Toulouse, ENSFEA, UT2J Toulouse, France

## Abstract

Monte Carlo is famous for accepting model extensions and model refinements up to infinite dimension. However, this powerful incremental design is based on a premise which has severely limited its application so far: a state-variable can only be recursively defined as a function of underlying state-variables if this function is linear. Here we show that this premise can be alleviated by projecting nonlinearities onto a polynomial basis and increasing the configuration space dimension. Considering phytoplankton growth in light-limited environments, radiative transfer in planetary atmospheres, electromagnetic scattering by particles, and concentrated solar power plant production, we prove the real-world usability of this advance in four test cases which were previously regarded as impracticable using Monte Carlo approaches. We also illustrate an outstanding feature of our method when applied to acute problems with interacting particles: handling rare events is now straightforward. Overall, our extension preserves the features that made the method popular: addressing nonlinearities does not compromise on model refinement or system complexity, and convergence rates remain independent of dimension.

## Introduction

The standard Monte Carlo (MC) method is a technique to predict a physical observable by numerically estimating a statistical expectation over a multi-dimensional configuration space^1^. The reason why this method is so popular in all fields of scientific research is its intuitive nature. In general, simulation tools are designed in direct relation to the physical phenomena present in each discipline, and later refinements are gradual and straightforward. Model refinements merely extend sampling to other appropriate dimensions. The method is nonetheless mathematically rigorous: specialists specify observables that are implicitly translated into integral quantities which are estimated using random sampling in each direction of the configuration space. This statistical approach is highly powerful because the algorithm can be designed directly from the description of the system, whether it is deterministic or not, with no reworking or approximation.

**Figure 1:**
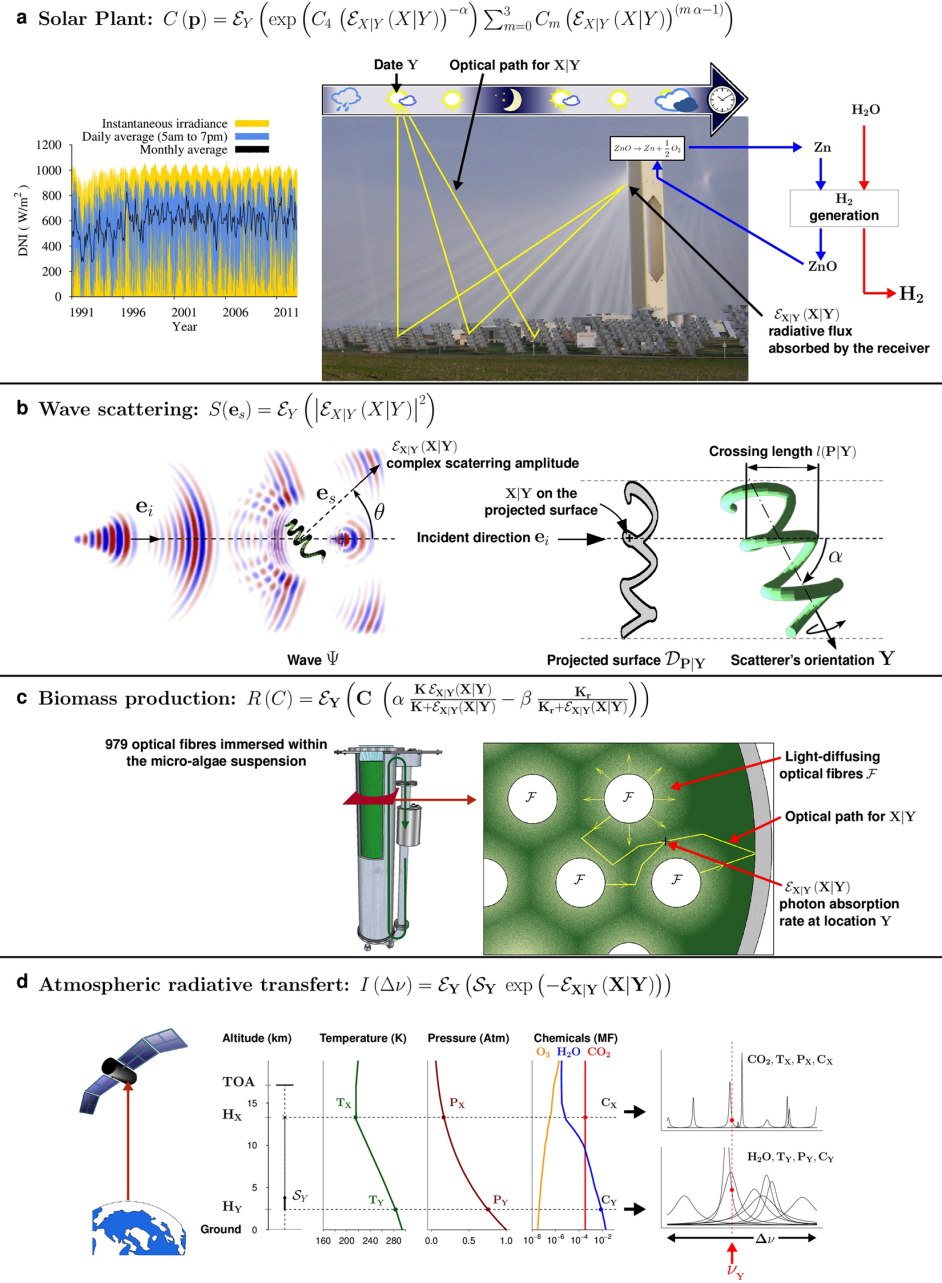
Complex systems with nonlinear outputs: four real-world examples. (**a**) Solar-driven high-temperature thermal reduction of zinc oxide, as the first phase of a two-step water-splitting cycle. Photons emitted from the sun are reflected on heliostats and concentrated at the entrance of the chemical reactor in which *ZnO* dissociation is carried out. Depending on their transmission success **X**, the solar power ${ {\mathcal E} }_{{\bf{X}}|{\bf{Y}}}({\bf{X}}|{\bf{Y}})$ absorbed by the receiver at a random instant **Y** determines the nonlinear chemical conversion rate of the reaction $ZnO\to Zn+\frac{1}{2}O$. Here we address the estimation of the solar plant’s annual conversion rate *C*(**p**) at different Earth locations **p**, by averaging the instantaneous conversion rates over the statistics of sun position and incident Direct Normal Irradiance (DNI), which fluctuates with time and weather conditions (see also SI1). (**b**) Wave scattering by a complex-shaped and optically soft scatterer (cyanobacterium *Arthrospira*). An incident plane wave with propagation direction **e**_*i*_ is scattered by the helical cyanobacterium. The bacterium has low relative refractive index and is much larger than the wavelength (optically soft particle). The complex scattering amplitude ${ {\mathcal E} }_{{\bf{X}}|{\bf{Y}}}({\bf{X}}|{\bf{Y}})$ in the forward directions is the sum of secondary wave contributions **X**|**Y** (interference) originating from the scatterer surface. This surface depends on the scatterer orientation **Y**. Here we address the estimation of *S*(**e**_*s*_), the single-scattering differential cross-section, in direction **e**_*s*_ for a suspension of particles, assuming independent scattering, by averaging the squared modulus of ${ {\mathcal E} }_{{\bf{X}}|{\bf{Y}}}({\bf{X}}|{\bf{Y}})$ over the statistics of orientations **Y** (see also SI2). (**c**) Phytoplankton growth in light-limited environments. Phytoplankton is grown in a continuous stirred tank photobioreactor, internally illuminated by optical fibres ${\mathcal F}$ immersed in the culture. The local rate of photon absorption ${ {\mathcal E} }_{{\bf{X}}|{\bf{Y}}}({\bf{X}}|{\bf{Y}})$ at location **Y** is the average of the contributions **X**|**Y** of every optical path from the fibres to **Y** through the scattering and absorbing suspension. ${ {\mathcal E} }_{{\bf{X}}|{\bf{Y}}}({\bf{X}}|{\bf{Y}})$ determines the nonlinear photosynthetic growth rate at location **Y**. Here we address the Monte Carlo estimation of *R*(*C*), the global growth-rate in the whole culture volume, as a function of biomass concentration *C*, by averaging the local rate over locations in the volume (see also SI3). (**d**) Atmospheric radiative transfer: top-of-atmosphere (TOA) specific intensity (from earth towards outer space). Photons emitted by the atmosphere at infrared frequencies are due to random emission transitions **Y**, from a higher to a lower energy state, of mainly CO_2_ and H_2_O molecules of concentration *C*_**Y**_ at altitude *H*_**Y**_. The corresponding source ${{\mathscr{S}}}_{{\bf{Y}}}$ depends on the thermodynamic state of the atmosphere, mainly temperature *T*_**Y**_ (defining the energy-state population) and pressure *P*_**Y**_ (defining most of the line width, i.e. the uncertainty of the emission frequency *ν*_**Y**_). This source is then exponentially attenuated by atmospheric absorption, i.e. by all random absorption transitions **X**|**Y**, from a lower to a higher energy state, occurring at altitude *H*_**X**|**Y**_ between *H*_**Y**_ and the top of the atmosphere (see also SI4). Copyright. “Central solar PS10” (https://commons.wikimedia.org/wiki/File:Luz.jpg) by MwAce is released into the public domain. “satellite” (www.pixabay.com/en/satellite-solar-panels-space-297840), “blue-earth” (pixabay.com/en/earth-blue-land-globe-planet-297125), “forecast icons” (www.pixabay.com/en/weather-signs-symbols-forecast-28719) and “time-clock” (pixabay.com/en/clock-time-hour-minute-wall-clock-295201) by Clker-Free-Vector-Images (www.pixabay.com/en/users/Clker-Free-Vector-Images-3736) are licensed under CC0 Creative Commons (www.creativecommons.org/publicdomain/zero/1.0/deed.en).

Let us illustrate how MC is used in engineering with a typical example: the optimal design of a concentrated solar plant^2^ (see Fig. 1a). The power collected by the central receiver results from all the rays of sunlight that reach it after reflection by heliostats, so it depends on the complex geometry of the heliostats. Moreover, the heliostats change their orientation to follow the sun’s position, so they can mask one another at certain times of the day. To estimate by MC the received power at one moment of interest, i.e. for a given geometry of the heliostats: choose an optical path among those that link the sun to the central receiver via a heliostat; check whether this path is shadowed or blocked by another heliostat; and retain a Monte Carlo weight equal to 0 or 1 depending on transmission success. Let **X** be the random variable denoting transmission success. The collected fraction of the available sun power is then the expectation ${ {\mathcal E} }_{{\bf{X}}}({\bf{X}})$ of **X**, and can be evaluated with no bias as the average of such weights over a large number of sampled paths.

This approach robustly complies with expanded descriptions of the physical observable to be addressed. For instance, the fraction of the available sun power collected on average over the entire lifetime of the solar plant (typically 30 years) can be predicted as the expectation over time of ${ {\mathcal E} }_{{\bf{X}}}({\bf{X}})$, which varies with time. Denoting ${ {\mathcal E} }_{{\bf{X}}|{\bf{Y}}}({\bf{X}}|{\bf{Y}})$ the collected fraction at random time **Y** within the 30 years, the time-averaged fraction is given by ${ {\mathcal E} }_{{\bf{Y}}}({ {\mathcal E} }_{{\bf{X}}|{\bf{Y}}}({\bf{X}}|{\bf{Y}}))={ {\mathcal E} }_{{\bf{Y}},{\bf{X}}|{\bf{Y}}}({\bf{X}}|{\bf{Y}})$. The basic algorithm above can then be encapsulated within time sampling: first choose a date for **Y**, then pick a path at that date for **X**|**Y**. Finally, estimate ${ {\mathcal E} }_{{\bf{Y}},{\bf{X}}|{\bf{Y}}}({\bf{X}}|{\bf{Y}})$ by computing the average transmission success over all combined pairs (date, path). Meanwhile, sun power fluctuations can be accounted for by estimating the atmospheric transmission at each chosen date. The choice of the statistical viewpoint thus enables us to incorporate into one single statistical question as many elements as necessary: the geometrical complexity of the heliostats^3^, the daily course of the sun, and seasonal-scale as well as hourly-scale weather fluctuations^4^. Remarkably, the latter question is nearly as simple to address as the estimation of the power collected at one single date: the algorithmic design can map the full conceptual description, yet computational costs are hardly affected. Contrastingly, deterministic approaches would translate into impractical computation times or require simplified and approximate descriptions, so MC has become the only practical solution in many engineering contexts of this type. Having become standard practice, MC has prompted numerous theoretical developments^5–8^.

Nevertheless, MC has so far not been able to handle *every* question. In fact, it was identified early on that “the extension of Monte Carlo methods to nonlinear processes may be impossible”^9^ and it is a prevalent opinion nowadays that “Monte Carlo methods are not generally effective for nonlinear problems, mainly because expectations are linear in character”^10^, so that “a nonlinear problem must usually be linearized in order to use the Monte Carlo technique”^11^. We are aware of only one attempt so far to bypass this failing: the recent proposal by the applied mathematics community^1,12–14^ to use branching processes^15^ to solve Fredholm-type integral equations with polynomial nonlinearity.

Unfortunately, most real-world problems are nonlinear. Indeed, if the question were now to evaluate the final return on investment of the solar plant, namely how much electrical power it would deliver over its lifetime, standard MC would fail, because the instantaneous conversion efficiency from collected solar power to electrical power is not linear. Let us consider, as a toy example, a basic nonlinear case where the electrical power would be proportional to the square of the instantaneous collected solar power ${ {\mathcal E} }_{{\bf{X}}|{\bf{Y}}}({\bf{X}}|{\bf{Y}})$ at date **Y**. In Monte-Carlo terms, the question would then be to estimate ${ {\mathcal E} }_{{\bf{Y}}}({ {\mathcal E} }_{{\bf{X}}|{\bf{Y}}}{({\bf{X}}|{\bf{Y}})}^{2})$ over the plant’s lifetime. In this case, the optical and temporal expectations can no longer be combined, because it would be wrong to first estimate, as above, the total solar power collected over its lifetime, and then apply the conversion efficiency at the end (basically, ${ {\mathcal E} }_{{\bf{Y}}}({ {\mathcal E} }_{{\bf{X}}|{\bf{Y}}}{({\bf{X}}|{\bf{Y}})}^{2})\ne { {\mathcal E} }_{{\bf{Y}}}{({ {\mathcal E} }_{{\bf{X}}|{\bf{Y}}}({\bf{X}}|{\bf{Y}}))}^{2}$, in the same way as *a*^2^ + *b*^2^ ≠ (*a* + *b*)^2^). Instead, we would have to sample dates (say *M* dates, millions over 30 years), estimate the solar power collected at each date by averaging transmission successes over numerous optical paths (say *N* paths, millions for each date), apply a nonlinear conversion to the result at that date, and then average over all dates^16^. Doing so, MC would now require *M* × *N* samples, and even worse, further levels of complexity (each adding a nonlinearity to the problem) would similarly multiply the computation time. Moreover, the result would be biased due to the finite sampling sizes of the innermost dimensions. In short, MC’s distinctive features are no longer available, and exact lifetime integration appears impossible.

Bearing in mind our earlier theoretical works about MC integral formulations^2^, we have found a way to bypass this obstacle for a large class of nonlinear problems, based on the very statistical nature of MC. In the case of our toy example, we use the fact that:

1 $${ {\mathcal E} }_{{\bf{Y}}}({ {\mathcal E} }_{{\bf{X}}|{\bf{Y}}}{({\bf{X}}|{\bf{Y}})}^{2})={ {\mathcal E} }_{{\bf{Y}},({{\bf{X}}}_{{\bf{1}}},{{\bf{X}}}_{{\bf{2}}})|{\bf{Y}}}({{\bf{X}}}_{{\bf{1}}}\,{{\bf{X}}}_{{\bf{2}}}|{\bf{Y}})$$

where **X**_1_ and **X**_2_ are two independent variables, identically distributed as **X** (see Methods). Translated into a sampling algorithm, the solution is now to sample optical paths in pairs (**X**_**1**_, **X**_**2**_)|**Y** (instead of millions) at each sampled date, and then to retain the pair product **X**_**1**_**X**_**2**_|**Y** of their transmission successes. The optical and temporal statistics can then actually be sampled together, and yield the unbiased result with no combinatorial explosion. This reformulation can be generalised to any nonlinearity of polynomial shape. First, monomials of any degree can indeed be estimated using the same sampling property as that used above for *n* = 2:

2 $${ {\mathcal E} }_{{\bf{Y}}}({ {\mathcal E} }_{{\bf{X}}|{\bf{Y}}}{({\bf{X}}|{\bf{Y}})}^{n})={ {\mathcal E} }_{{\bf{Y}},({{\bf{X}}}_{{\bf{1}}},{{\bf{X}}}_{{\bf{2}}},\ldots ,{{\bf{X}}}_{{\bf{n}}})|{\bf{Y}}}({{\bf{X}}}_{{\bf{1}}}\,{{\bf{X}}}_{{\bf{2}}}\ldots {{\bf{X}}}_{{\bf{n}}}|{\bf{Y}})$$

where **X**_*i*_ are *n* independent random variables, identically distributed as **X**. For any monomial of degree *n*, the expectation can then be computed by sampling series of *n* independent realisations of **X**|**Y**, and averaging the series products. The linear case, solved by standard MC, corresponds to *n* = 1. Secondly, since polynomials are simply linear combinations of monomials, the expectation for any polynomial function of ${ {\mathcal E} }_{{\bf{X}}|{\bf{Y}}}({\bf{X}}|{\bf{Y}})$ of degree *n* can be translated into a Monte Carlo algorithm, first sampling a degree in the polynomial, and then sampling as many independent realisations of **X**|**Y** as this random degree (see *Methods*). For a polynomial function of degree *n*, the corresponding Non-Linear Monte Carlo (NLMC) algorithm is then:

- pick a sample *y* of **Y**,
- choose a monomial degree value *d* ≤ *n*,
- draw *d* independent samples of **X**|**Y** = **y** and retain their product,

repeat this sampling procedure and compute the estimate as the average of the retained products.

Moreover, if polynomial forms of any dimension are now solvable with no approximation, so is the projection of any nonlinear function onto a polynomial basis of any dimension, even of infinite dimension if required (full details of using the Taylor expansion are given in *Methods*). As a result, any hierarchy of nested statistical processes that combine nonlinearly can now, in theory, be exactly addressed within the Monte Carlo framework. The deep rationale of the proposed algorithm is therefore to transform a nonlinear process into a formally equivalent linear infinite-dimension process, and then use the inherent capability of Monte Carlo to address expectations over domains of infinite dimension.

To the best of our knowledge, this analysis has never before been performed. However, it has major practical consequences for real-world problems, provided the polynomial sampling, which is the price to be paid for tackling nonlinearities exactly, remains tractable. For instance, let us go back to our solar power plant example, and now use the actual expression for the conversion rate and its Taylor expansion: for each date, once a sun position and climate conditions have been fixed, we would have to pick a random number of independent optical paths (instead of one optical path in the linear case), keep the product of their transmission success, and finally calculate the average of many such products. Doing so, it becomes possible to integrate hourly solar input fluctuations over 30 years in the full geometry of a kilometre-wide heliostat field in order to optimise the nonlinear solar-to-electric conversion over the plant lifetime (Fig. 1a). The same line of thought can be used to predict wave scattering by a tiny complex-shaped scatterer^17^ such as a helicoidal cyanobacterium (Fig. 1b). The biomass production example (Fig. 1c), where incoming light favours the photosynthetic growth that in turn blocks the incoming light, illustrates how our method also handles nonlinear feedback^18^. Finally, with the estimation of Earth’s radiative cooling (Fig. 1d), we reproduce quite classic results, yet with a purely statistical approach: by sampling directly the state transitions of greenhouse gases, we avoid costly deterministic computations that the standard linear Monte Carlo approach requires in order to by-pass the nonlinearity of the Beer Extinction Law^19^. In each of the four cases, it appears that the additional computations are well affordable using only ordinary computing power (the complete physical descriptions of the four problems, the nonlinearities involved and their translation in NLMC can be found in their respective Extended Data Figures and Supplemental Information, Solar Plant: SI1; Complex-shaped Scatterer: SI2; Biomass production: SI3; Earth radiative cooling: SI4).

For these four real-world simulation examples, we can therefore retain that the variance of the proposed statistical estimate was very much satisfactory. Is that a general feature? Can we feel confident when applying this simulation strategy to any new nonlinear problem? More generally speaking, what do we claim about the status of the present research? Essentially, we only argue that the general proposition of the present paper is immediately available for an ensemble of pratical applications. Indeed, these four simulation examples are representative of a quite wide ensemble of physics/engineering practices and the corresponding implementations are now practically used by the corresponding research-communities^17,19–21^. Moreover, implementation only required an up to date knowledge of Monte Carlo techniques: the probability sets were selected using nothing more than very standard importance-sampling reasoning (see Methods, SI1 and SI3). Outside these experiments, we did not explore in any systematic manner the statistical convergence difficulties that could be specifically associated to the proposition. But although we did not yet encounter it, we can already point out a potential source of variance related to the choice of the fixed point *x*_0_ around which the nonlinear function is Taylor expanded (see Methods).

From a theoretical point of view, in the four cases exposed above, the model is directly enunciated in statistical terms, defining two random variables **X** and **Y** from the start. More broadly, standard MC practice can also start from a deterministic description (see Methods), most commonly from a linear partial differential equation (PDE). The formal equivalence between the solution of a linear PDE and the expectation of a random variable has long been established^22^. Indeed, PDE-to-MC translations are essential to nanoscale mechanics (Quantum Monte Carlo^23^) or nuclear sciences. NLMC allows such translations for nonlinear PDEs.

As an illustration of the ground-breaking nature of our study, we address a prominent example of a nonlinear PDE in statistical physics, the Boltzmann equation, which governs the spatiotemporal density of interacting particles in a dilute gas (full details in SI5). The corresponding physics is easy to visualise: a particle simply follows its ballistic flight until it collides with another particle. The collisions are considered as instantaneous and only modify the two particle velocities. The equation for the variation in particle density in phase-space (position, velocity) is nonlinear because the collision rate depends on the density itself. In order to project this nonlinearity onto the proper polynomial basis of infinite dimension, this PDE is first translated into its Fredholm integral counterpart (a step reminiscent of the aforementioned Dimov proposition^1^). This Fredholm integral expresses the density in phase space at some location for some velocity at some time, as if putting a probe into space-time. It is estimated by Monte Carlo, tracking the dynamics backwards in time up to the initial condition (or boundary conditions). Importantly, such a probe estimation does not require the exhaustive resolution of the whole field at previous times: as in standard backwards MC algorithms for solving linear transport (e.g. simulating an image by tracking photon-paths backward, from receiver to source^24–26^) the information about previous states of the field is reconstructed along each path only where and when it is required^27^. Here, the contrast with linear MC is that nonlinearity due to collisions translates into branching paths.

**Figure 2:**
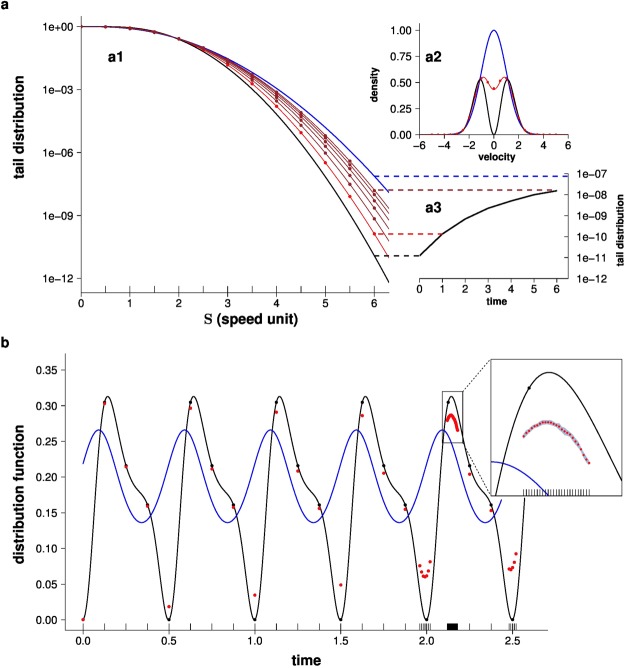
Nonlinear Monte Carlo for gas kinetics. (**a**) Relaxation of speed distribution to equilibrium ((a1) tail distribution of particle speeds (fraction of particles faster than **S**), (a2) probability density function of velocity, (a3) variation in the fraction of particles faster than 6 speed units). In a homogenous gas, collisions between particles redistribute velocities so that the speed distribution tends to equilibrium (Maxwellian distribution). Starting from a distribution far from equilibrium (black curves), we compute the relaxation to a Maxwellian distribution (blue curves) by estimating the tail distribution at different times (e.g. red curves correspond to the system state at 1 unit time). The continuous lines correspond to the analytical solutions and each point corresponds to an independent NLMC estimation. The fraction of particles which are faster than 6 speed units (a3) illustrates how NLMC well accounts for the 1000-fold increase in the rarest high-speed particles, with no space or time discretisation. Remarkably, rare events are estimated with the same relative uncertainty as frequent events (10^4^ samples for each estimate; confidence intervals of all estimates are contained within the thickness of the point). (**b**) Dampening of breathing mode. A dilute gas confined by an outside harmonic potential displays a *breathing mode*. We estimate the density at probe position (1.75, 0, 0) and velocity (0.35, 0, 0) (in adimensional units, see SI5) at different times (adim. unit). Starting from a distribution complying with the local equilibrium, the density displays a perpetual oscillation at twice the trap frequency (blue curve, analytical solution), independent of the collision rate (or, equivalently, of the elastic cross section). Starting from a distribution which is far from the local equilibrium (the same initial distribution as in (a)), the density still pulsates in the absence of collisions (null cross section; black curve: analytical solution; black points: probe estimates). When collisions are introduced (by raising the cross section), the velocity redistribution induced by the collisions dampens the oscillation (red points: probe estimates; no analytical solution). The inset illustrates that probes can be bunched together to zoom in on a period of special interest (e.g. estimating the peak values at each cycle). Each point is estimated independently using 10^7^ samples; confidence intervals of all estimates are contained within the thickness of one point in the main figure, and confidence intervals are represented by the grey background area in the inset).

This extension deals very efficiently with extremely rare events because it preserves an essential feature of MC: by avoiding time/space/velocity discretisation^28–30^, very low densities can be estimated with no bias, and the only source of uncertainty is the finite number of sampled events (i.e. the confidence interval around the estimated density). As a test, we consider a case for which analytical solutions have been published: Krook’s early analysis of the distribution of speeds in extremely out-of-equilibrium conditions^31,32^. Krook’s analysis was outstanding because it provided an analytical solution to a problem which looked impossible to solve numerically: events with the greatest consequences, namely the particles with the highest energies (i.e. high-speed particles, of tremendous importance in nuclear chemistry) lie far out in the tail of speed distribution and have a very low probability of occurrence (rare events). Using our NLMC design, the fractions of particles which have a kinetic energy higher than 10^6^ times the average value, and which correspond to a fraction as low as 10^−11^ of the total, can now be quantified as accurately as desired, and perfectly fit the analytical solution (Fig. 2a).

Having been validated in Krook’s case, this extension opens the way to solving systems for which no analytical solutions are available. As an example, we now consider a fully spatialised system in which the particles are confined by an outside harmonic potential, leading to a so-called *breathing mode* of the gas density. Such a solution to the Boltzmann equation was identified early on by Boltzmann himself ^33^, but has recently been revisited and generalised in the context of a shortcut to adiabaticity techniques for classic gases^34^. Exact solutions are available only under the constraint that the gas is at local equilibrium, in which case the density displays a permanent oscillation. Here again, these analytical solutions are exactly recovered. Moreover, NLMC enables us to go beyond this constraint and to explore the gas behaviour when the local equilibrium constraint is alleviated: starting from a state far from local equilibrium, it is now possible to estimate how fast the velocity redistribution induced by collisions actually dampens the oscillation (Fig. 2b).

## Conclusions

From now on, the Monte Carlo Method is no longer restricted to linear problems. The five examples exposed above were worked out by teams comprising specialists of the Monte Carlo method and specialists of the physical problem under consideration. Through their complete description, we offer readers all the details to implement their own applications. As a guideline, the first step is to formulate the physical observable under its expectation form, including the nonlinearities and integrating all levels of complexity. The second step is to reformulate this expectation as a formulation compliant with the standard Monte Carlo Method, according to the type of nonlinearity. For polynomial nonlinearities, use i.i.d. series products. For other differentiable forms, use a Taylor expansion around an upper bound of the innermost random variable in order to regain a polynomial form. Using this MC-compliant formulation, every advanced MC technique can then be applied: parallel implementation, complex geometry, null collisions, zero variance, control variate, importance sampling, sensitivity analysis, and so on. As illustrated by the variety of our seminal examples, this guideline covers a large set of nonlinear academic and real-world problems.

## Methods

### Basics of Monte Carlo Methods

Let us estimate *E* = 1 + 4 by repeatedly tossing a (fair) coin. The tossing process is described by a random variable *R* ∈ {0, 1} which takes the value 0 for heads (probability ${P}_{R}(0)=\tfrac{1}{2}$) and 1 for tails (probability ${P}_{R}(1)=\tfrac{1}{2}$).

Now, to estimate any process (e.g. a process output: *E* = 1 + 4), we can assign arbitrary weights *w*(*R*) to values {0, 1} in order to write *E* as an expectation of the weighted process, following:

3 $${E}=1+4={P}_{R}(0)w(0)+{P}_{R}(1)w(1)={ {\mathcal E} }_{R}(w(R))$$

with $w(0)=\tfrac{1}{{P}_{R}(0)}=2$ and $w(1)=\tfrac{4}{{P}_{R}(1)}=8$ and where ${ {\mathcal E} }_{R}$ denotes the expectation with respect to *R*. Using the results *r*_1_ … *r*_*N*_ of *N* successive tosses (independent realisations of *R*), we can then estimate $E={ {\mathcal E} }_{R}(w(R))$ from the weighted average of the toss results $\frac{1}{N}\,{\sum }_{i=1}^{N}\,w({r}_{i})$ since *E* = 5 is indeed the average of Monte Carlo weights that take the values 2 and 8 with equal probabilities.

Such an approach is at the base of Monte Carlo techniques: define the weights according to the problem to be solved, sample the process repeatedly, and take the average. Depending on the physical description of the value to be estimated, this approach still holds for an infinite number of terms and can also be extended to integral formulation using continuous random variables:

4 $${{\rm{E}}}_{{\bf{Y}}}(w({\bf{Y}}))={\int }_{{{\mathscr{D}}}_{{\bf{Y}}}}\,dy\,{p}_{{\bf{Y}}}(y)\,w(y)$$

which can be estimated by $\frac{1}{N}\,{\sum }_{i=1}^{N}\,w({{\bf{y}}}_{i})$, where the *y*_*i*_ are *N* realisations of the random variable *Y* with probability density function *p*_*Y*_ and domain of definition ${{\mathscr{D}}}_{{\bf{Y}}}$.

### Basics of Nonlinear Monte Carlo Methods

In order to estimate

5 $${\rm{E}}={{\mathscr{E}}}_{{\bf{Y}}}({{\mathscr{E}}}_{{\bf{X}}|{\bf{Y}}}{({\bf{X}}|{\bf{Y}})}^{2})={\int }_{{{\mathscr{D}}}_{{\bf{Y}}}}\,dy\,{p}_{{\bf{Y}}}(y)\,{({\int }_{{{\mathscr{D}}}_{{\bf{X}}|{\bf{Y}}}}dx{p}_{{\bf{X}}|{\bf{Y}}}(x|y)x)}^{2}$$

we introduce two independent variables **X**_1_ and **X**_2_, identically distributed as **X** (still conditioned by the same **Y**):

6 $$\begin{array}{ccc}{\rm{E}} & = & {{\mathscr{E}}}_{{\bf{Y}}}({{\mathscr{E}}}_{{{\bf{X}}}_{{\bf{1}}}|{\bf{Y}}}({{\bf{X}}}_{{\bf{1}}}|{\bf{Y}})\,{{\mathscr{E}}}_{{{\bf{X}}}_{{\bf{2}}}|{\bf{Y}}}({{\bf{X}}}_{{\bf{2}}}|{\bf{Y}}))\\  & = & {\int }_{{{\mathscr{D}}}_{{\bf{Y}}}}\,dy\,{p}_{{\bf{Y}}}(y)\,({\int }_{{{\mathscr{D}}}_{{\bf{X}}|{\bf{Y}}}}\,d{x}_{1}\,{p}_{{\bf{X}}|{\bf{Y}}}({x}_{1}|y)\,{x}_{1})\,({\int }_{{{\mathscr{D}}}_{{\bf{X}}|{\bf{Y}}}}\,d{x}_{2}\,{p}_{{\bf{X}}|{\bf{Y}}}({x}_{2}|y)\,{x}_{2})\end{array}$$

Since **X**_1_ and **X**_2_ are independent, and conditionally independent given **Y**:

7 $$\begin{array}{ccc}{\rm{E}} & = & {\int }_{{{\mathscr{D}}}_{{\bf{Y}}}}\,dy\,{p}_{{\bf{Y}}}(y)\,({\iint }_{{{\mathscr{D}}}_{{\bf{X}}|{\bf{Y}}}^{2}}\,d{x}_{1}\,{p}_{{\bf{X}}|{\bf{Y}}}({x}_{1}|y)\,d{x}_{2}\,{p}_{{\bf{X}}|{\bf{Y}}}({x}_{2}|y)\,{x}_{1}{x}_{2})\\  & = & {{\mathscr{E}}}_{{\bf{Y}}}({{\mathscr{E}}}_{({{\bf{X}}}_{{\bf{1}}},{{\bf{X}}}_{{\bf{2}}})|{\bf{Y}}}({{\bf{X}}}_{{\bf{1}}}{{\bf{X}}}_{{\bf{2}}}|{\bf{Y}}))\end{array}$$

Hence

8 $$\begin{array}{ccc}{\rm{E}} & = & {\iiint }_{{{\mathscr{D}}}_{{\bf{Y}}}\times {{\mathscr{D}}}_{{\bf{X}}|{\bf{Y}}}^{2}}\,dy\,{p}_{{\bf{Y}}}(y)\,d{x}_{1}\,{p}_{{\bf{X}}|{\bf{Y}}}({x}_{1}|y)\,d{x}_{2}\,{p}_{{\bf{X}}|{\bf{Y}}}({x}_{2}|y)\,{x}_{1}{x}_{2}\\  & = & {{\mathscr{E}}}_{{\bf{Y}},({{\bf{X}}}_{{\bf{1}}},{{\bf{X}}}_{{\bf{2}}})|{\bf{Y}}}({{\bf{X}}}_{{\bf{1}}}{{\bf{X}}}_{{\bf{2}}}|{\bf{Y}})\end{array}$$

The same demonstration can be made to establish that:

9 $${ {\mathcal E} }_{{\bf{Y}}}\,({ {\mathcal E} }_{{\bf{X}}|{\bf{Y}}}{({\bf{X}}|{\bf{Y}})}^{n})={ {\mathcal E} }_{{\bf{Y}},({{\bf{X}}}_{{\bf{1}}},{{\bf{X}}}_{{\bf{2}}},\ldots ,{{\bf{X}}}_{{\bf{n}}})|{\bf{Y}}}({{\bf{X}}}_{{\bf{1}}}\,{{\bf{X}}}_{{\bf{2}}}\ldots {{\bf{X}}}_{{\bf{n}}}|{\bf{Y}})$$

Let us now assume that the weights associated with the random variable **Y** are described by a nonlinear function **f**(**Z**_**Y**_) of the conditional expectation ${{\bf{Z}}}_{{\bf{Y}}}={ {\mathcal E} }_{{\bf{X}}|{\bf{Y}}}({\bf{X}}|{\bf{Y}})$. The problem then becomes to compute:

10 $$E={{\mathscr{E}}}_{{\bf{Y}}}({\bf{f}}({{\bf{Z}}}_{{\bf{Y}}}))={{\mathscr{E}}}_{{\bf{Y}}}({\bf{f}}({{\mathscr{E}}}_{{\bf{X}}|{\bf{Y}}}({\bf{X}}|{\bf{Y}})))$$

Such a nonlinearity can be treated with no approximation using a projection on an infinite basis. In all the examples presented in this article, we have used a Taylor polynomials basis, which means that **f**(*x*) is expanded around *x*_0_

11 $${\bf{f}}(x)=\sum _{n=0}^{+\infty }\,\frac{{\partial }^{n}{\bf{f}}({x}_{0})}{n!}{(x-{x}_{0})}^{n}$$

We note that both *x*_0_ and **f** can be conditioned by **Y**. Now, following the same line as explained above for the Basics of Monte Carlo Methods, we regard the sum in the expansion of **f** as an expectation, writing:

12 $${\bf{f}}(x)={{\mathscr{E}}}_{N}(\frac{{{\rm{\partial }}}^{N}{\bf{f}}({x}_{0})}{{P}_{N}(N)N!}{(x-{x}_{0})}^{N})$$

where the random variable *N* (of probability law *P*_*N*_) is the degree of one monomial in the Taylor polynomial. This step only requires us to define one infinite set of probabilities (instead of two in Eq. 3), with ${\sum }_{n=0}^{+\infty }\,{P}_{N}(n)=1$.

Equation 10 can then be rewritten as:

13 $$E={{\mathscr{E}}}_{{\bf{Y}}}({\bf{f}}({{\mathscr{E}}}_{{\bf{X}}|{\bf{Y}}}({\bf{X}}|{\bf{Y}})))={{\mathscr{E}}}_{Y,N}(\frac{{{\rm{\partial }}}^{N}{\bf{f}}({x}_{0})}{{P}_{N}(N)N!}{({{\mathscr{E}}}_{{\bf{X}}|{\bf{Y}}}({\bf{X}}|{\bf{Y}})-{x}_{0})}^{N})$$

Defining independent and identically distributed random variables **X**_*q*_, with the same distribution as **X**, the innermost term rewrites

14 $$E={{\mathscr{E}}}_{Y,N}(\frac{{{\rm{\partial }}}^{N}{\bf{f}}({x}_{0})}{{P}_{N}(N)N!}\,\prod _{q=1}^{N}\,({{\mathscr{E}}}_{{{\bf{X}}}_{{\bf{q}}}|{\bf{Y}}}({{\bf{X}}}_{{\bf{q}}}|{\bf{Y}})-{x}_{0}))$$

or, equivalently:

15 $$E={{\mathscr{E}}}_{Y,N}(\frac{{{\rm{\partial }}}^{N}{\bf{f}}({x}_{0})}{{P}_{N}(N)N!}\,\prod _{q=1}^{N}\,{{\mathscr{E}}}_{{{\bf{X}}}_{{\bf{q}}}|{\bf{Y}}}({{\bf{X}}}_{{\bf{q}}}|{\bf{Y}}-{x}_{0}))$$

Since the variables **X**_*q*_|**Y** are independent in the innermost term, we have:

16 $$\prod _{q=1}^{N}\,{{\mathscr{E}}}_{{{\bf{X}}}_{{\bf{q}}}|{\bf{Y}}}({{\bf{X}}}_{{\bf{q}}}|{\bf{Y}}-{x}_{0})={{\mathscr{E}}}_{({{\bf{X}}}_{{\bf{1}}},{{\bf{X}}}_{{\bf{2}}},\ldots ,{{\bf{X}}}_{{\bf{N}}})|{\bf{Y}}}(\prod _{q=1}^{N}\,({{\bf{X}}}_{q}|{\bf{Y}}-{x}_{0}))$$

so that:

17 $$E={{\mathscr{E}}}_{{\bf{Y}},{\bf{N}}}(\frac{{{\rm{\partial }}}^{N}{\bf{f}}({x}_{0})}{{P}_{N}(N)N!}\,{{\mathscr{E}}}_{({{\bf{X}}}_{{\bf{1}}},{{\bf{X}}}_{{\bf{2}}},\ldots ,{{\bf{X}}}_{{\bf{N}}})|{\bf{Y}}}(\prod _{q=1}^{N}\,({{\bf{X}}}_{q}|{\bf{Y}}-{x}_{0})))$$

and we finally have:

18 $$E={{\mathscr{E}}}_{{\bf{Y}},{\bf{N}},({{\bf{X}}}_{{\bf{1}}},{{\bf{X}}}_{{\bf{2}}},\ldots ,{{\bf{X}}}_{{\bf{N}}})|{\bf{Y}}}(\frac{{{\rm{\partial }}}^{N}{\bf{f}}({x}_{0})}{{P}_{N}(N)N!}\,\prod _{q=1}^{N}\,({{\bf{X}}}_{q}|{\bf{Y}}-{x}_{0}))$$

which can be read as:

19 $$E={{\mathscr{E}}}_{{\bf{Y}},{\bf{N}},({{\bf{X}}}_{{\bf{1}}},{{\bf{X}}}_{{\bf{2}}},\ldots ,{{\bf{X}}}_{{\bf{N}}})|{\bf{Y}}}(w({\bf{Y}},{\bf{N}},{{\bf{X}}}_{{\bf{1}}},{{\bf{X}}}_{{\bf{2}}},\ldots ,{{\bf{X}}}_{{\bf{N}}}))$$

with

20 $$w({\bf{Y}},{\bf{N}},{{\bf{X}}}_{{\bf{1}}},{{\bf{X}}}_{{\bf{2}}},\ldots ,{{\bf{X}}}_{{\bf{N}}})=\frac{{\partial }^{N}{\bf{f}}({x}_{0})}{{P}_{N}(N)N!}\,\prod _{q=1}^{N}\,({{\bf{X}}}_{q}|{\bf{Y}}-{x}_{0})$$

With the notation above, ${\prod }_{q=1}^{0}\,({{\bf{X}}}_{q}|{\bf{Y}}-{x}_{0})=1$.

The translation into a Monte Carlo algorithm then follows:

- sample a realisation *y* of **Y** (and set *x*_0_ and **f** accordingly if they depend on y)
- sample a realisation *n* of *N*
- sample *n* independent realisations *x*_*q*=1,…,*n*_ of **X** conditioned by y
- keep

$$\hat{w}=w(y,n,{x}_{1},\ldots ,{x}_{n})=\frac{{{\rm{\partial }}}^{n}{\bf{f}}({x}_{0})}{{P}_{N}(n)n!}\,\prod _{q=1}^{n}\,({x}_{q}-{x}_{0})$$

and estimate *E* as the average of many realisations $\hat{w}$.

### Implementation example

Let us illustrate the choice of the discrete distribution *P* on *N* with an implementation example. We take *Y* uniformly distributed over [0, 1], *X*|*Y* uniformly distributed over [0, *Y*] and *f*(*x*) = 1/(1 + *x*) (*f* corresponds to the photobioreactor real-world example in Fig. 1c, with *C* = 1, *α* = 0, *β* = −1, *K*_*r*_ = 1). Equation 10 becomes

21 $$E={{\mathscr{E}}}_{Y}(\frac{1}{1+{{\mathscr{E}}}_{X|Y}(X|Y)})$$

Its analytical solution is *E* = 2 ln(3/2).

Injecting the n-th derivative ${{\rm{\partial }}}^{n}f({x}_{0})=\frac{n!\,{(-1)}^{n}}{{(1+{x}_{0})}^{n+1}}$ into Equation 20 leads to

$$w(Y,N,{X}_{1},{X}_{2},\ldots ,{X}_{N})=\frac{{(-1)}^{N}}{{P}_{N}(N)\,{(1+{x}_{0})}^{N+1}}\,\prod _{q=1}^{N}\,({X}_{q}|Y-{x}_{0})$$

that can be reformulated as

22 $$w(Y,N,{X}_{1},{X}_{2},\ldots ,{X}_{N})=\frac{1}{{P}_{N}(N)}\frac{{x}_{0}^{N}}{{(1+{x}_{0})}^{N+1}}\,\prod _{q=1}^{N}\,\frac{{X}_{q}|Y-{x}_{0}}{{x}_{0}}$$

Using standard importance-sampling reasoning, we choose the set of probabilities that cancels the term $\frac{{x}_{0}^{N}}{{(1+{x}_{0})}^{N+1}}$ in the estimator:

23 $$w(Y,N,{X}_{1},{X}_{2},\ldots ,{X}_{N})=\prod _{q=1}^{N}\frac{{X}_{q}|Y-{x}_{0}}{{x}_{0}}$$

with

24 $${P}_{N}(N)=\frac{{x}_{0}^{N}}{{(1+{x}_{0})}^{N+1}}$$

The NLMC algorithm is

- sample a realisation *y* of *Y*
- sample a realisation *n* of *N* according to the discrete distribution in Equation 24
- sample *n* independent realisations *x*_*q*=1,…,*n*_ of *X* uniformly distributed over [0, *y*]
- keep

$$\hat{w}=\prod _{q=1}^{n}\,\frac{{x}_{q}-{x}_{0}}{{x}_{0}}$$

and estimate *E* as the average of *M* realisations $\hat{w}$.

**Figure 3:**
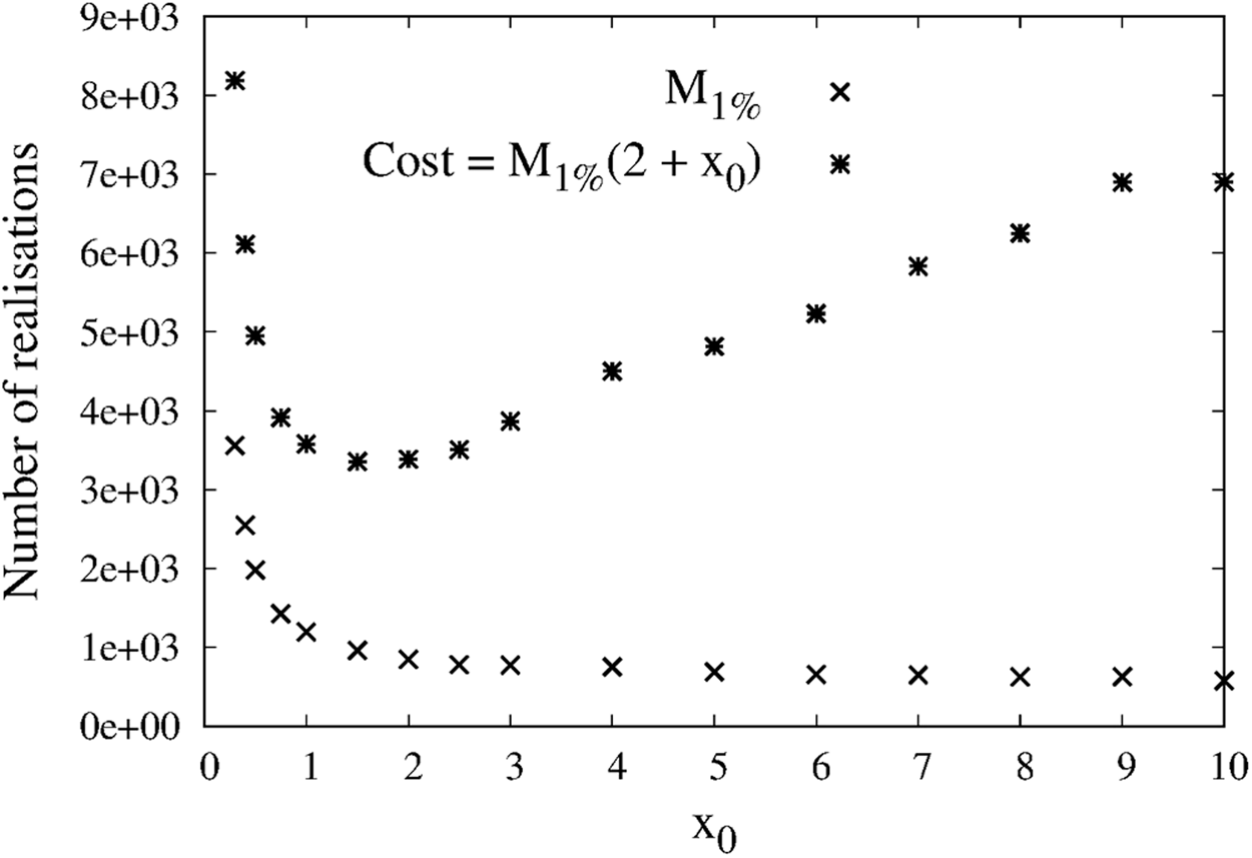
Choice of the discrete distribution *P* on *N*: a tradeoff between estimation and computational cost. Estimation of equation 21 with the estimator in equation 23, as a function of the choice of the fixed point *x*_0_ around which the nonlinear function is Taylor expanded. In this example, *x*_0_ also defines the discrete distribution *P*_*N*_ (see equation 24). Crosses indicate the number of realisations *M*_1%_ that are required to achieve an estimation with 1% standard deviation. Stars indicate the corresponding computational cost as defined in equation 25.

We define the computational cost *C* of this algorithm in terms of the total number of random generations that are required to achieve 1% standard deviation on the estimation. Each realisation of the algorithm includes 1 random generation *y* of *Y*, 1 random generation *n* of *N*, and *n* random generations of *X*. And it takes *M*_1%_ realisations of the algorithm to achieve a standard deviation of 1%. Overall,

25 $$C={M}_{1 \% }(2+ {\mathcal E} (N))={M}_{1 \% }(2+{x}_{0})$$

since ${\mathcal E} (N)={x}_{0}$ with the discrete distribution in Equation 24. Figure 3 shows the values of *M*_1%_ and *C* recorded with simulations, as a function of *x*_0_. The choice of *x*_0_ alone controls both the statistical convergence (*i*.*e*. *M*_1%_) and the computational cost (through the discrete distribution *P* on *N*). We observe a trade-off between estimation and computational cost. For low values of *x*_0_, only few realisations of *X* are needed since the discrete distribution on *N* is rapidly decreasing with *n*, but a large number of realisation of the algorithm are required for the estimation (*i*.*e*. ${\mathcal E} (N)$ is small but *M*_1%_ is large). At the opposite, for larger values of *x*_0_, the estimator converges rapidly, but the average number of *X* random generations per Monte Carlo realisations is increased (*i*.*e*. *M*_1%_ is small but ${\mathcal E} (N)$ is large). In between, we observe an optimal choice of *x*_0_.

### Comparison with the naive plug-in estimator and convergence issues

In the previous implementation example solving equation 21, a naive plug-in estimator could be constructed as^16^:

$$E={{\mathscr{E}}}_{Y}(\frac{1}{1+\frac{1}{K}\,{\sum }_{q=1}^{K}\,{X}_{q}|Y})$$

leading to the following Monte Carlo algorithm:

- sample a realisation *y* of *Y*
- sample *K* independent realisations *x*_*q*=1,…,*K*_ of *X* uniformly distributed over [0, *y*]
- keep

$$\hat{w}=\frac{1}{1+\frac{1}{K}\,{\sum }_{q=1}^{K}\,{x}_{q}}$$

and estimate *E* as the average of *M* realisations $\hat{w}$. With this algorithm, *K* must ensure that the bias of the estimator can be neglected. For that purpose, we choose the value *K*_1%_ that always gives an estimation of ${ {\mathcal E} }_{X|Y}$ with 1% standard deviation. Therefore, each realisation of the algorithm includes 1 random generation for *Y* and *K*_1%_ generations for *X*, and it takes *M*_1%_ realisations of the algorithm to achieve a standard deviation of 1% on the estimation of *E*. The computational cost of the naive algorithm is therefore *M*_1%_(1 + *K*_1%_). In the present example, *K*_1%_ = 3333 and we observed that *M*_1%_ = 140 thanks to numerical simulations: the cost is *C* = 466760. Compared to the results in Fig. 3, even for this very simple example where *Y* and *X*|*Y* have little variance, the computational cost of the naive plug-in algorithm is 100 times higher than that of the NLMC estimator. Nevertheless, this conclusion only stands for a reasonable choice of *x*_0_ (and therefore of *P*_*N*_). Indeed, the computational cost with the NLMC estimator seems to rise up to infinity when *x*_0_ approaches 0 (see Equation 23 and Fig. 3): even the naive plug-in algorithm would then be a better choice. Although we did not further analyse this observation with theoretical means, we can at least retain that chosing *x*_0_ is likely to be an essential step of the present approach as far as computational costs are concerned.

## Electronic supplementary material

Supplemental Information

## References

[1] Dimov, I. T. & McKee, S. Monte Carlo Methods for Applied Scientists (World Scientific Publishing, 2008).

[2] Delatorre J (2014). Monte Carlo advances and concentrated solar applications. Sol. Energy.

[3] Siala FMF, Elayeb ME (2001). Mathematical formulation of a graphical method for a no-blocking heliostat field layout. Renew. energy.

[4] Farges O (2015). Life-time integration using Monte Carlo Methods when optimizing the design of concentrated solar power plants. Sol. Energy.

[5] Assaraf R, Caffarel M (1999). Zero-variance principle for Monte Carlo algorithms. Phys. Rev. Lett..

[6] Metropolis N, Rosenbluth AW, Rosenbluth MN, Teller AH, Teller E (1953). Equation of state calculations by fast computing machines. J. Chem. Phys..

[7] Hammersley JM, Handscomb DC (1964). Monte Carlo Methods.

[8] Roger M, Blanco S, El Hafi M, Fournier R (2005). Monte Carlo Estimates of Domain-Deformation Sensitivities. Phys. Rev. Lett..

[9] Curtiss JH (1953). ‘Monte Carlo’ Methods for the Iteration of Linear Operators. J. Math. Phys..

[10] Kalos MH, Whitlock PA (2008). Monte Carlo Methods.

[11] Chatterjee K, Roadcap JR, Singh S (2014). A new Green’s function Monte Carlo algorithm for the solution of the two-dimensional nonlinear Poisson–Boltzmann equation: Application to the modeling of the communication breakdown problem in space vehicles during re-entry. J. Comput. Phys..

[12] Vajargah BF, Moradi M (2007). Monte Carlo algorithms for solving Fredholm integral equations and Fredholm differential integral equations. Appl. Math. Sci..

[13] Rasulov A, Raimova G, Mascagni M (2008). Monte Carlo solution of Cauchy problem for a nonlinear parabolic equation. Math. Comput. Simulation.

[14] Gobet, E. Monte-Carlo Methods and Stochastic Processes: From Linear to Non-linear (CRC Press, 2016).

[15] Skorokhod AV (1964). Branching diffusion processes. Theory Probab. Appl..

[16] Hong, L. J. & Juneja, S. Estimating the Mean of a Non-linear Function of Conditional Expectation. Proceedings of the 2009 Winter Simulation Conference, Austin, Texas, 1223–1236 (2009).

[17] Charon J (2016). Monte Carlo implementation of Schiff’s approximation for estimating radiative properties of homogeneous, simple-shaped and optically soft particles: Application to photosynthetic micro-organisms. J. Quant. Spectrosc. Radiat. Transf..

[18] Cornet JF (2010). Calculation of optimal design and ideal productivities of volumetrically lightened photobioreactors using the constructal approach. Chem. Eng. Sci..

[19] Galtier M (2016). Radiative transfer and spectroscopic databases: A line-sampling Monte Carlo approach. J. Quant. Spectrosc. Radiat. Transf..

[20] Dauchet, J. *et al*. Calculation of the radiative properties of photosynthetic microorganisms. *J*. *Quant*. *Spectrosc*. *Radiat*. *Transfer*. (2015).

[21] Dauchet J, Cornet J-F, Gros F, Roudet M, Dussap C-G (2016). Chapter One – Photobioreactor Modeling and Radiative Transfer Analysis for Engineering Purposes. Adv. Chem. Eng..

[22] Kac, M. On some connections between probability theory and differential and integral equations. Proc. Second Berkeley Symp. Math. Statistics Probab. 189 (1951).

[23] Corney JF, Drummond PD (2004). Gaussian quantum Monte Carlo methods for fermions and bosons. Phys. Rev. Lett..

[24] Pharr, M. & Humphreys, G. Physically Based Rendering: from theory to implementation (Elsevier, 2010).

[25] Case KM (1957). Transfer problems and the reciprocity principle. Rev. Mod. Phys..

[26] Collins DG, Blättner WG, Wells MB, Horak HG (1972). Backward Monte Carlo calculations of the polarization characteristics of the radiation emerging from spherical-shell atmospheres. Appl. Opt..

[27] Galtier M (2013). Integral formulation of null-collision Monte Carlo algorithms. J. Quant. Spectrosc. Radiat. Transf..

[28] Wagner W (1995). Stochastic particle methods and approximation of the Boltzmann equation. Math. Comput. Simul..

[29] Rjasanow S (1996). A Stochastic Weighted Particle Method for the Boltzmann Equation. J. Comput. Phys..

[30] Rjasanow S, Wagner W (2001). Simulation of rare events by the stochastic weighted particle method for the Boltzmann equation. Math. Comput. Model..

[31] Krook M, Wu TT (1976). Formation of Maxwellian Tails. Phys. Rev. Lett..

[32] Krook M, Wu TT (1977). Exact solutions of the Boltzmann equation. Phys. Fluids.

[33] Boltzmann, L. In Wissenschaftliche Abhandlungen, edited by Hasenorl, F. Vol. II, p. 83 (J.A. Barth, Leipzig, 1909).

[34] Guéry-Odelin D, Muga JG, Ruiz-Montero MJ, Trizac E (2014). Nonequilibrium Solutions of the Boltzmann Equation under the Action of an External Force. Phys. Rev. Lett..

