# Supplementary material for: Addressing nonlinearities in Monte Carlo
Source: Sci Rep. 2018 Sep 5;8:13302. doi: 10.1038/s41598-018-31574-4 (PMC6125312; doi:10.1038/s41598-018-31574-4)
Supplement: Supplementary file 1 — Supplemental Information [file 41598_2018_31574_MOESM1_ESM.pdf]

Supplemental Information for article "Addressing Nonlinearities in Monte Carlo"

Jérémi Dauchet[1], Jean-Jacques Bezian[2], Stéphane Blanco[3], Cyril Caliot[4], Julien Charon[1], Christophe Coustet[5], Mouna El Hafi[2], Vincent Eymet[5], Olivier Farges[6], Vincent Forest[5], Richard Fournier[3], Mathieu Galtier[7], Jacques Gautrais[8], Anaïs Khuong[8], Lionel Pelissier[9], Benjamin Piaud[5], Maxime Roger[7], Guillaume Terrée[2] & Sebastian Weitz[1]

[1] *Université Clermont Auvergne, Sigma-Clermont, Institut Pascal, BP 10448, F-63000 Clermont-Ferrand, France.*

[2] *Université Fédérale de Toulouse Midi-Pyrénées, Mines Albi, UMR CNRS 5302, Centre RAPSODEE, Campus Jarlard, F-81013 Albi CT Cedex.*

[3] *LAPLACE, Université de Toulouse, CNRS, INPT, UPS, France.*

[4] *Processes, Materials and Solar Energy Laboratory, PROMES, CNRS, 7 rue du Four Solaire, 66120 Font-Romeu-Odeillo-Via, France.*

[5] *Méso-Star SAS, www.meso-star.com.*

[6] *Université de Lorraine, LEMTA, CNRS, UMR 7563, Vandoeuvre-lès-Nancy, F-54500, France*

[7] *Univ Lyon, CNRS, INSA-Lyon, Université Claude Bernard Lyon 1, CETHIL UMR5008, F-69621, Villeurbanne, France.*

[8] *Centre de Recherches sur la Cognition Animale, Centre de Biologie Intégrative (CBI), Centre National de la Recherche Scientifique (CNRS), Université Paul Sabatier (UPS), F-31062 Toulouse Cedex 9, France.*

[9] *Education, Formation, Travail, Savoirs (EFTS), Université de Toulouse, ENSFEA, UT2J, France.*

Contents

SI1 — Solar thermochemical reduction of zinc oxide averaged over the year **3**

SI2 — Wave scattering **7**

SI3 — Phytoplankton growth in light-limited environments **11**

SI4 — Atmospheric radiative transfer: top-of-atmosphere specific intensity (from earth toward the outer space) **15**

SI5 — Gas kinetics **19**

a

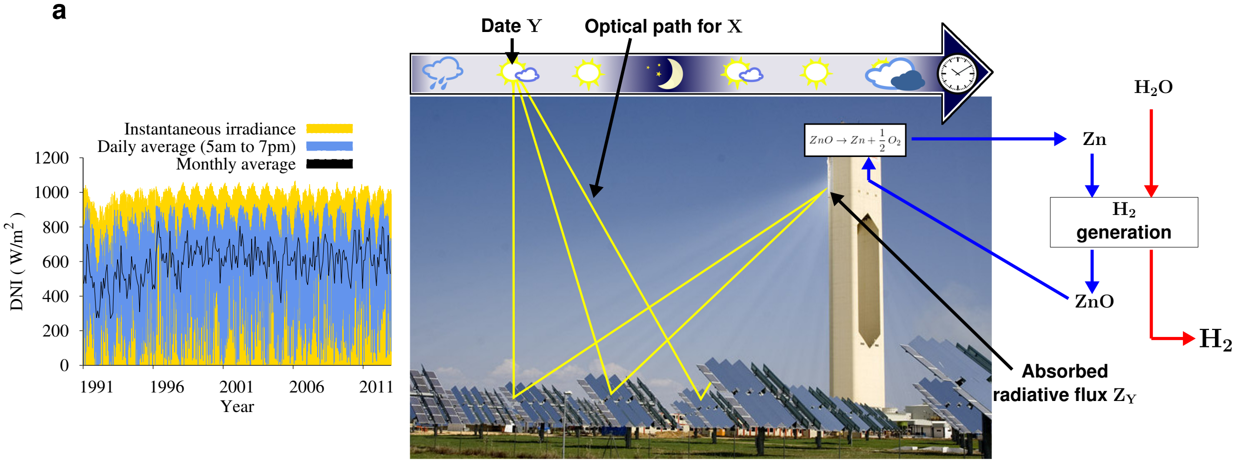

b

Time-average of chemical conversion rate at some earth location p

$$C\left(\mathbf{p}\right)=\mathcal{E}_{\mathbf{Y}}\left(\mathrm{f}\left(\mathbf{Z}_{\mathbf{Y}}\right)\right)$$

$\mathrm{f}\left(\mathbf{Z}_{\mathbf{Y}}\right)=\exp\left(C_4\left(\mathbf{Z}_{\mathbf{Y}}\right)^{-\alpha}\right)\sum_{m=0}^{3}C_m\left(\mathbf{Z}_{\mathbf{Y}}\right)^{(m\,\alpha-1)}$	Chemical conversion rate
$\mathbf{Z}_{\mathbf{Y}}=\mathcal{E}_{\mathbf{X}\mid\mathbf{Y}}\left(\mathbf{X}\mid\mathbf{Y}\right)$	Radiative power absorbed by the receiver
$\mathbf{Y}$	Date at the considered earth location, determining sun position and incoming solar power depending on wheather conditions
$\mathbf{X}\mid\mathbf{Y}$	Contribution at date Y of sun emission along one multiple-reflection optical path leading to the receiver

c

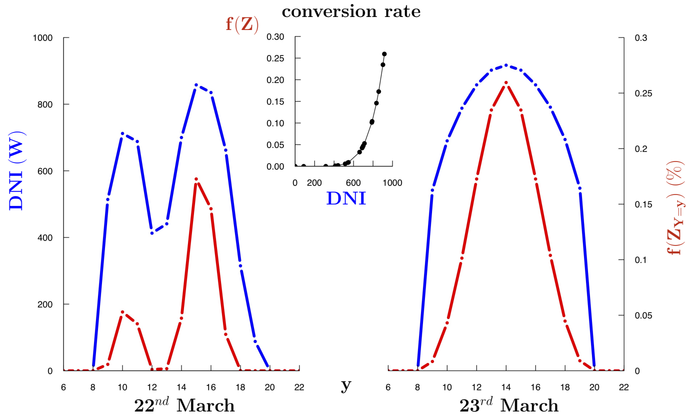

d

Location	$C\left(\mathbf{p}\right)$
Sevilla	0.87 ± 0.01
Paris	0.85 ± 0.01
Albuquerque	0.9 ± 0.01

Extended Data Figure 1 | Solar thermochemical reduction of zinc oxide: conversion rate over years. **a**, Solar-driven high temperature thermochemical cycles processes, commonly based on metal oxides reduction, are an alternative to fossil fuel-based method for H_2 generation. Their practical interest depends however upon their lifetime average productivity. Here we focus on thermal reduction of zinc oxide, as the first part of a two step water splitting cycle. Photons emitted from the sun are reflected on heliostats and concentrated at the entrance of the chemical reactor in which ZnO dissociation is carried out. Solar power $\mathbf{Z}_{\mathbf{Y}}$ absorbed by the reactor at a given instant $\mathbf{Y}$ of lifetime determines the non linear chemical conversion rate $\mathrm{f}\left(\mathbf{Z}_{\mathbf{Y}}\right)$ of the reaction $ZnO \rightarrow Zn + \frac{1}{2}O$. **b**, Here we address the estimation of the annual solar-plant's conversion rate $C(\mathbf{p})$ at different earth locations $\mathbf{p}$, by averaging the instantaneous conversion rate $\mathrm{f}\left(\mathbf{Z}_{\mathbf{Y}}\right)$ over the statistics of sun position and incident Direct Normal Irradiance (DNI, which fluctuates with weather). The Monte Carlo estimation combines the thermochemical knowledge of the non-linear kinetics of zinc oxide dissociation with the description of radiative transfer from the sun to a multiple-reflection central receiver solar plant. The instantaneous thermal-power $\mathbf{Z}_{\mathbf{Y}}$ collected by the receiver at moment $\mathbf{Y}$ of the year is the average of the contribution $\mathbf{X}|\mathbf{Y}$ of sun emissions along every optical paths. This solar power $\mathbf{Z}_{\mathbf{Y}}$ received at the entrance of the chemical reactor is used to reduce the zinc oxide with a thermochemical conversion rate $\mathrm{f}\left(\mathbf{Z}_{\mathbf{Y}}\right)$. **c**, Two typical days showing the received sun power (DNI, Direct Normal Irradiance) which yields $\mathbf{Z}_{\mathbf{Y}}$ after concentration by heliostats, and the conversion rate $\mathrm{f}\left(\mathbf{Z}_{\mathbf{Y}}\right)$; the corresponding conversion rate from DNI to $\mathrm{f}\left(\mathbf{Z}_{\mathbf{Y}}\right)$ is indicated in the inset. **d**, Estimated solar-plant's annual conversion rate in three earth locations.

SI1 — Solar thermochemical reduction of zinc oxide averaged over the year

The problem (see Extended Data Figure 1). Solar-driven high temperature thermochemical cycles processes, commonly based on metal oxides reduction [1,2], are an alternative to fossil fuel-based method for H_2 generation. Here we focus on thermal reduction zinc oxide, as the first part of a two step water splitting cycle. Photons emitted from the sun are reflected on heliostats and concentrated at the entrance of the chemical reactor in which ZnO dissociation is carried out. Solar power received by the reactor at a given instant determines the chemical conversion rate of the reaction $\mathrm{ZnO} \rightarrow \mathrm{Zn} + \frac{1}{2}\mathrm{O}$. The present Monte Carlo estimation of the solar-plant's annual conversion rate C associates the thermochemical knowledge of the non-linear kinetics of zinc oxide dissociation [3,4] to the description of radiative transfer on a multiple-reflection solar receiver [5]. The nonlinearity lies in the instantaneous coupling between photon transport and zinc-oxide reduction.

The random variable $\mathbf{X}$ is the contribution of an optical path from the sun to the entrance of the chemical reactor. Its expectation $\mathcal{E}_{\mathbf{X}|\mathbf{Y}}(\mathbf{X}|\mathbf{Y})$ is the instantaneous thermal-power fraction collected at a given moment $\mathbf{Y}$ of the year[6]. Solar power is used to reduce the zinc oxide with a nonlinear conversion rate $\mathrm{f}\left(\mathcal{E}_{\mathbf{X}|\mathbf{Y}}(\mathbf{X}|\mathbf{Y})\right)$ (see[3,4]).

Non-Linear Monte Carlo formulation The annual conversion rate C is reformulated as its Taylor expansion around x_0. First, independent and identically distributed (i.i.d) optical-path random variables are introduced, each defining a random contribution $\mathbf{X}_i|\mathbf{Y}$ i.i.d as $\mathbf{X}|\mathbf{Y}$. Then, we expand the non-linear function f around x_0 chosen as an upper bound of $\mathbf{X}|\mathbf{Y}$. Finally, the infinite power series is statistically formulated thanks to the discrete random variable N the order of Taylor expansion:

$$N_{\mathbf{Y}} = B_{\mathbf{X}_1|\mathbf{Y}} + \sum_{i=2}^{+\infty} i\, B_{\mathbf{X}_i|\mathbf{Y}} \prod_{q=1}^{i-1} (1 - B_{\mathbf{X}_q|\mathbf{Y}}) \tag{1}$$

where the $B_{\mathbf{X}_i|\mathbf{Y}}$ are Bernoulli random variables:

$$B_{\mathbf{X}_i|\mathbf{Y}} = \begin{cases} 1 & \text{with probability} \quad P_{\mathbf{X}_i|\mathbf{Y}} = \dfrac{\mathbf{X}_i|\mathbf{Y}}{x_0} \\ \\ 0 & \text{with probability} \quad 1 - P_{\mathbf{X}_i|\mathbf{Y}} \end{cases} \tag{2}$$

In the end, the reformulation is:

$$C = \mathcal{E}_{\mathbf{Y},(\mathbf{X}_1,\mathbf{X}_2,\ldots,\mathbf{X}_{\mathbf{N_Y}})|\mathbf{Y}}\left(w(N_{\mathbf{Y}})\right) \tag{3}$$

where the Monte Carlo weight function w is

$$w(u) = \begin{cases} \exp\left(C_4\, x_0^{-\alpha}\right) \displaystyle\sum_{m=1}^{3} C_m\, x_0^{(m\,\alpha-1)} \quad \text{if } u = 1 \\ \\ w(1) + \exp\left(C_4\, x_0^{-\alpha}\right) \displaystyle\sum_{k=0}^{u} (-1)^k \sum_{p=0}^{k} \frac{1}{p!(k-p)!} \sum_{m=1}^{3} C_m\, x_0^{(m\alpha-1+p)} \\ \qquad \times \displaystyle\prod_{j=1}^{k-p} (m\alpha - j) \sum_{r=1}^{p} (-1)^r\, C_4\, x_0^{-(\alpha+r)} \prod_{l=0}^{r-1} (\alpha + l) \quad \text{if } u > 1 \end{cases} \tag{4}$$

with $u \in \mathbb{N}$ and C_1, C_2, C_3, C_4, α and x_0 known constants.

Algorithm

Step 1 Uniform sampling of a moment $\mathbf{y}$ of the year and index initialisation $n = 1$.

Step 2 Sampling of the n-th optical path contributing to thermal-power collection at instant $\mathbf{y}$ and computation of its contribution x_n (as presented in[5,6]).

Step 3 Computation of the probability P_n in Eq. 2 and sampling of a realisation b_n of the Bernoulli random variable $B_{\mathbf{X}_n|\mathbf{Y}}$.

Step 4 If $b_n = 0$, the algorithm loops to step 2 after incrementation of n. Else, the procedure is terminated and the weight $\hat{w}(n)$ computed according to Eq. 4.

Simulated configuration Results shown in Extended Data Figure 1 are obtained for a 1 MW solar plant with a 80 m high central receiver tour and 160 heliostats arranged in a radial stagered layout (nueen method). Values of the conversion-rate parameters C_1, C_2, C_3, C_4 and α are given in [3,4]. Meteonorm DNI database.

a

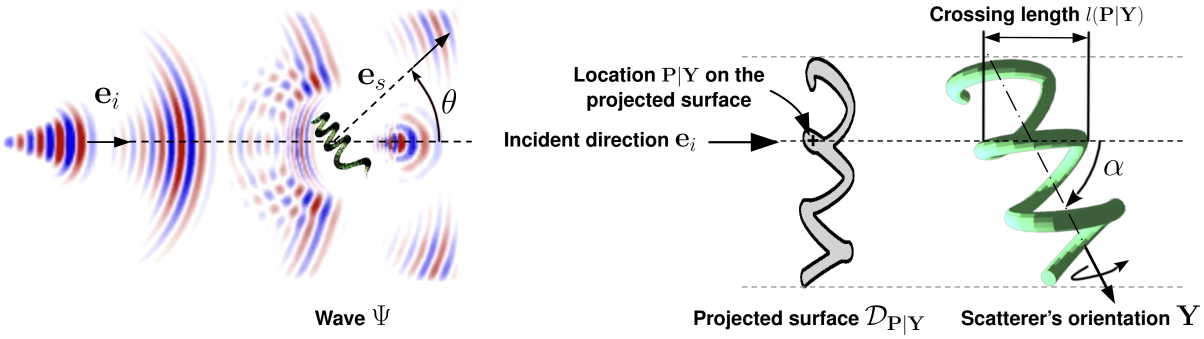

b

Differential scattering cross-section averaged over scatterer's orientations

$$W(\mathbf{e}_s) = \mathcal{E}_{\mathbf{Y}}\left(f(\,S_{\mathbf{Y}}\,)\right)$$

$f(S_{\mathbf{Y}}) = \lvert S_{\mathbf{Y}} \rvert^2$	**Differential scattering cross-section**
$S_{\mathbf{Y}} = \mathcal{E}_{X\lvert\mathbf{Y}}\left(X\lvert\mathbf{Y}\right)$	**Complex scattering amplitude**
$\mathbf{Y}$	**Scatterer orientation with respect to $\mathbf{e}_i$**
$X\lvert\mathbf{Y}$	**Contribution of the scattering potential through point P of the projected surface**

c **d**

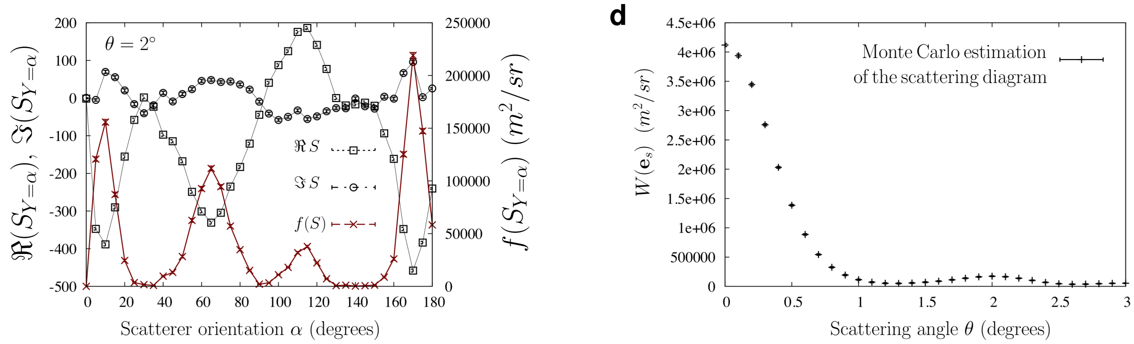

Extended Data Figure 2 | Wave scattering by a complex-shaped and optically-soft scatterer (cyanobacterium *Arthrospira*). **a**, An incident plane wave with propagation direction $\mathbf{e}_i$ and wave number k is scattered by the cyanobacterium. The cyanobacterium is large compared to $1/k$ and has low relative refractive index (optically-soft scatterer). Therefore, the scalar wave approximation is used : the field Ψ resulting from the interaction between the incident wave and the scattering potential U is solution of the scalar wave equation $(\nabla^2 + k^2 - U)\Psi = 0$. This wave scattering problem is identical to that of high-energy potential scattering studied by L.I. Schiff in the context of quantum mechanics. Under Schiff's approximation, the complex scattering-amplitude $S_{\mathbf{Y}}$ (far-field region) in the forward directions depends on the scatterer orientation $\mathbf{Y}$ through its projected surface on the plane of the incident direction $\mathbf{e}_i$. **b**, Here we address the estimation of $W(\mathbf{e}_s)$ the single-scattering differential cross-section in direction $\mathbf{e}_s$ for a scatterer ensemble, by averaging the differential scattering cross-section $f(S_{\mathbf{Y}}) = |S_{\mathbf{Y}}|^2$ over the statistics of orientations $\mathbf{Y}$ (independent scattering regime). The Monte Carlo estimation of $W(\mathbf{e}_s) = \mathcal{E}_{\mathbf{Y}}\left(f(S_{\mathbf{Y}})\right)$ at small scattering angles θ combines the description of waves Ψ propagation with the non-linear formulation of the power that they carry $|\Psi|^2$ (Poynting vector magnitude). The scattering amplitude $S_{\mathbf{Y}}$ results from the interference of secondary waves contributions $\mathbf{X}|\mathbf{Y}$ originating from the projected surface of the scatterer with orientation $\mathbf{Y}$. This scattering amplitude determines the differential cross-section f$(S_{\mathbf{Y}})$ for that orientation. **c**, For a given direction $\mathbf{e}_s$ ($\theta = 2°$), the scatterer orientation can greatly affect the wave shape, resulting in huge variations of the transmitted power after scattering (e.g. by five orders of magnitude from $\alpha = 140°$ to $\alpha = 170°$). **d**, After averaging over an isotropic orientation distribution, the cross-section depends only on θ.

SI2 — Wave scattering

This example is fully detailed in Charon *et al.* 2016[7].

The problem (see Extended Data Figure 2). Here we address the solution of Schiff's approximation[8], also known as the *anomalous diffraction approximation*[9], for an incident plane wave with propagation direction $\mathbf{e}_i$ and wave number k scattered by the scattering potential U that takes value U^{in} inside a large domain compared to $1/k$ and 0 outside (this domain corresponds to the shape of the scatterer), with $|U^{in}| \ll k^2$. Let $\Gamma_{\mathbf{Y}}$ be the projected surface of the scatterer seen from $\mathbf{e}_i$, given the scatterer orientation $\mathbf{Y}$. Straight rays along $\mathbf{e}_i$ cross $\Gamma_{\mathbf{Y}}$ at locations $\mathbf{P}|\mathbf{Y}$; the domain of definition of $\mathbf{P}|\mathbf{Y}$ is then $\mathcal{D}_{\mathbf{P}|\mathbf{Y}} \equiv \mathbf{P}|\mathbf{Y}$. These rays are attenuated and phase shifted over the crossing length $l(\mathbf{P}|\mathbf{Y})$. The complex random variable $X|\mathbf{Y} = X_{\mathbf{P}|\mathbf{Y}}(\mathbf{e}_s)$ is the contribution of one of these rays to the scattered field in a direction $\mathbf{e}_s$ deviating from $\mathbf{e}_i$ by a small scattering angles θ[7]:

$$X_{\mathbf{P}|\mathbf{Y}}(\mathbf{e}_s) = A_{\mathbf{Y}} \frac{k}{2\,\pi} \exp(ik\,\theta\,\mathbf{b}\cdot\mathbf{P}|\mathbf{Y}) \left\{ 1 - \exp\left(\frac{i}{2k} U^{in}\, l(\mathbf{P}|\mathbf{Y}) \right) \right\} \tag{5}$$

where $\mathbf{b}$ is the unit vector along the projection of $\mathbf{e_s}$ on the plane containing $\mathcal{D}_{\mathbf{P}|\mathbf{Y}}$ and $A_{\mathbf{Y}}$ is the area of $\mathcal{D}_{\mathbf{P}|\mathbf{Y}}$. Given the orientation $\mathbf{Y}$ of the scatterer, the conditional expectation $S_{\mathbf{Y}}(\mathbf{e}_s) = \mathcal{E}_{X|\mathbf{Y}}(X|\mathbf{Y}) = \mathcal{E}_{\mathbf{P}|\mathbf{Y}}(X_{\mathbf{P}|\mathbf{Y}}(\mathbf{e}_s))$ is the complex scattering amplitude in direction $\mathbf{e}_s$[7–10]. For the scatterer orientation $\mathbf{Y}$, the far-field scattering diagram is given by the *differential scattering cross-section*[9,12]: $\hat{W}_{\mathbf{Y}}(\mathbf{e}_s) = |S_{\mathbf{Y}}(\mathbf{e}_s)|^2 = |\mathcal{E}_{\mathbf{P}|\mathbf{Y}}(X_{\mathbf{P}|\mathbf{Y}}(\mathbf{e}_s))|^2$.

We address the Monte Carlo computation of $W(\mathbf{e}_s) = \mathcal{E}_{\mathbf{Y}}(\hat{W}_{\mathbf{Y}}(\mathbf{e}_s))$ which is $\hat{W}_{\mathbf{Y}}(\mathbf{e}_s)$ averaged over the statistics of the scatterer orientation $\mathbf{Y}$ [9,10,12]. The full expression is then

$$\begin{aligned} W(\mathbf{e}_s) &= \mathcal{E}_{\mathbf{Y}}\left(|S_{\mathbf{Y}}(\mathbf{e}_s)|^2\right) \\ &= \mathcal{E}_{\mathbf{Y}}\left(|\mathcal{E}_{\mathbf{P}|\mathbf{Y}}(X_{\mathbf{P}|\mathbf{Y}}(\mathbf{e}_s))|^2\right) \\ &= \mathcal{E}_{\mathbf{Y}}\left(\left(\Re\,\mathcal{E}_{\mathbf{P}|\mathbf{Y}}(X_{\mathbf{P}|\mathbf{Y}}(\mathbf{e}_s))\right)^2 + \left(\Im\,\mathcal{E}_{\mathbf{P}|\mathbf{Y}}(X_{\mathbf{P}|\mathbf{Y}}(\mathbf{e}_s))\right)^2\right) \end{aligned} \tag{6}$$

where the configuration spaces are the orientation vectors of the scattering potential with respect to $\mathbf{e}_i$ ($\mathcal{D}_{\mathbf{Y}}$) and the projected surface of the scattering potential seen from the incident direction $\mathbf{e}_i$ ($\mathcal{D}_{\mathbf{P}|\mathbf{Y}}$).

Non-Linear Monte Carlo formulation $W(\mathbf{e}_s)$ is reformulated based on the definition of two independent and identically distributed location random-variables $\mathbf{P}_1|\mathbf{Y}$ and $\mathbf{P}_2|\mathbf{Y}$:

$$\begin{aligned} W(\mathbf{e}_s) &= \mathcal{E}_{\mathbf{Y}}\left(\Re\,\mathcal{E}_{\mathbf{P}_1|\mathbf{Y}}(X_{\mathbf{P}_1|\mathbf{Y}}(\mathbf{e}_s))\,\Re\,\mathcal{E}_{\mathbf{P}_2|\mathbf{Y}}(X_{\mathbf{P}_2|\mathbf{Y}}(\mathbf{e}_s)) + \Im\,\mathcal{E}_{\mathbf{P}_1|\mathbf{Y}}(X_{\mathbf{P}_1|\mathbf{Y}}(\mathbf{e}_s))\,\Im\,\mathcal{E}_{\mathbf{P}_2|\mathbf{Y}}(X_{\mathbf{P}_2|\mathbf{Y}}(\mathbf{e}_s))\right) \\ &= \mathcal{E}_{\mathbf{Y},(\mathbf{P}_1,\mathbf{P}_2)|\mathbf{Y}}\left(w(\mathbf{P}_1,\mathbf{P}_2,\mathbf{e}_s)\right) \end{aligned} \tag{7}$$

where the Monte Carlo weight function w is:

$$w(\mathbf{P}_1,\mathbf{P}_2,\mathbf{e}_s) = \Re\,X_{\mathbf{P}_1|\mathbf{Y}}(\mathbf{e}_s)\,\Re\,X_{\mathbf{P}_2|\mathbf{Y}}(\mathbf{e}_s) + \Im\,X_{\mathbf{P}_1|\mathbf{Y}}(\mathbf{e}_s)\,\Im\,X_{\mathbf{P}_2|\mathbf{Y}}(\mathbf{e}_s) \tag{8}$$

with $X_{\mathbf{P}_q|\mathbf{Y}}(\mathbf{e}_s)$ defined in Eq. 5.

Algorithm The sampling procedures of the Monte-Carlo algorithm are then:

Step 1 Isotropic sampling of an orientation $\mathbf{y}$ of the scattering potential.

Step 2 Uniform sampling of the first location $\mathbf{p}_1$ on the projected surface defined by $\mathbf{y}$ and computation of the corresponding crossing length $l(\mathbf{p}_1)$: the realisation x_1 of $X_{\mathbf{P}|\mathbf{Y}}(\mathbf{e}_s)$ is computed according to Eq. 5.

Step 3 Uniform sampling of the second location $\mathbf{p}_2$ on the projected surface defined by $\mathbf{y}$ and computation of the corresponding crossing length $l(\mathbf{p}_2)$: the realisation x_2 of $X_{\mathbf{P}|\mathbf{Y}}(\mathbf{e}_s)$ is computed according to Eq. 5.

Step 4 Computation of the weight $\hat{w} = w(\mathbf{p}_1,\mathbf{p}_2,\mathbf{e}_s)$ according to Eq. 8: $\hat{w} = \Re\,x_1\ \Re\,x_2 + \Im\,x_1\,\Im\,x_2$

Codes for the implementation of this algorithm in the case of spheroidal and cylindrical scattering potentials, as well as validation against reference solution, are provided in Charon et al. 2016 [7] (see also http://edstar.lmd.jussieu.fr/codes).

Simulated configuration Results shown in Extended Data Figure 2 are obtained for $k = 2\pi/\lambda$, with wavelength $\lambda = 500nm$ and the scattering potential $U^{in} = -2k(m-1)$, with $m = 1.2 - i\,4\,.10^{-3}$ (scatterer relative refractive index[10]). The shape of the scatterer is helical with length $L = 50\mu m$, pitch $P = 15\mu m$, helix diameter $D = 20\mu m$, cylinder diameter $d = 3.5\mu m$.

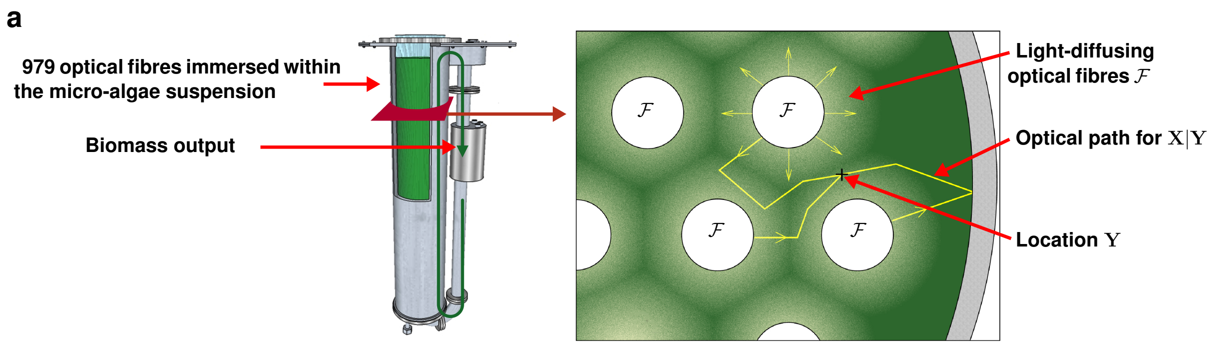

b

Biomass growth-rate averaged over the culture volume for biomass concentration C

$$R(C) = \mathcal{E}_{\mathbf{Y}}(\mathrm{f}\,(\mathbf{A}_{\mathbf{Y}}))$$

$\mathrm{f}(\mathbf{A}_{\mathbf{Y}}) = C\left(\alpha\,\frac{K\,\mathbf{A}_{\mathbf{Y}}}{K+\mathbf{A}_{\mathbf{Y}}} - \beta\,\frac{K_r}{K_r+\mathbf{A}_{\mathbf{Y}}}\right)$	local biomass growth rate for light input $\mathbf{A}_{\mathbf{Y}}$
$\mathbf{A}_{\mathbf{Y}} = \mathcal{E}_{\mathbf{X}\|\mathbf{Y}}\,(\mathbf{X}\|\mathbf{Y})$	rate of photon absorption by microalgae at location $\mathbf{Y}$
$\mathbf{Y}$	location within the volume
$\mathbf{X}\|\mathbf{Y}$	contribution to $\mathbf{A}_{\mathbf{Y}}$ along one probabilistic multiple-scattering optical path from fibers to location $\mathbf{Y}$

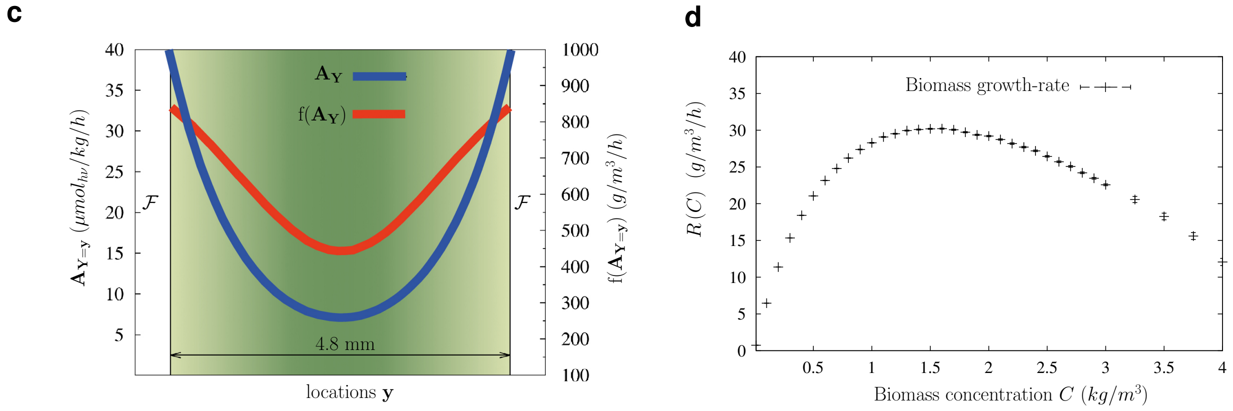

Extended Data Figure 3 | Phytoplankton growth in light-limited environments. a, Phytoplankton is put to grow in a continuous stirred tank photobioreactor, an enclosed and perfectly controlled environment insuring optimal pH and temperature conditions, as well as non-limiting CO_2 and minerals supplies to micro-algae: photosynthesis is only light-limited. Light is provided by 979 light-diffusing optical fibres $\mathcal{F}$ immersed within the phytoplankton culture. The fibres insure a quasi-uniform light-flux density on the totality of their surface. This diluted light-input triggers an artificially sustained algal bloom with high photosynthetic efficiency and high biomass growth-rate R. The local rate of photon absorption $A_{\mathbf{Y}}$ at location $\mathbf{Y}$ determines the non-linear photosynthetic-response $\mathrm{f}(A_{\mathbf{Y}})$ of cells at that location. **b**, Here, we address the Monte Carlo estimation of $R(C)$ the biomass growth-rate in the culture volume as a function of biomass concentration C, by averaging the local growth-rate $f(A_{\mathbf{Y}})$ over locations in the volume. The Monte Carlo estimation of $R(C) = \mathcal{E}_{\mathbf{Y}}(\mathrm{f}\,(\mathbf{A}_{\mathbf{Y}}))$ combines the available knowledge of the non-linear photosynthetic growth-rate of a single cell to the description of radiative transfer within the multiple-scattering and absorbing micro-algae suspension with concentration C. The rate of photon absorption $A_{\mathbf{Y}}$ at location $\mathbf{Y}$ is the average of the contributions $\mathbf{X}|\mathbf{Y}$ of every multiple-scattering optical paths from fibres to $\mathbf{Y}$. **c**, Photons absorbed by a phytoplankton-cell are non linearly converted within the photosynthetic units and the Z-scheme, leading to a spatial profile of the biomass growth-rate $\mathrm{f}(A_{\mathbf{Y}})$. **d**, The full-tank growth-rate is shown to depend upon the biomass concentration and indicates the optimal concentration allowing the largest biomass production rate. We note that $R(C)$ is usually denoted by $< r_x >$ (C_x) in the photobioreactor literature.

SI3 — Phytoplankton growth in light-limited environments

The problem (see Extended Data Figure 3). Here we address the production of reference solutions for a photobioreactor model. This model is based on a radiative transfer approach presented in[11]. The rate of photon absorption $\mathbf{A_Y}$ by micro-algae at location $\mathbf{Y}$ within the culture volume is solution of the Radiative Transfer Equation. The present study is based on the standard linear transport MC algorithm presented in[15] for the estimation of $\mathbf{A_Y}$ at any location within the photobioreactor. In this algorithm, realisations of the optical path random variable $\mathbf{\Gamma}(C)|\mathbf{Y}$ are sampled backward from the absorption location $\mathbf{Y}$ to the light emitting surface (the surface of the 979 light-diffusing optical fibres, see EDF3). Since micro-algae are scattering visible light, $\mathbf{\Gamma}(C)|\mathbf{Y}$ depends on their concentration C within the suspension: the scattering and absorption coefficient of the suspension are proportional to C (independent scattering). The random variable $\mathbf{X}(C)|\mathbf{Y} = \mathbf{X}_{\mathbf{\Gamma}(C)|\mathbf{Y}}(C)$ is the contribution of one of these optical paths to the photon absorption rate at $\mathbf{Y}$ (see[15] for the detailed expression of $\mathbf{X}_{\mathbf{\Gamma}(C)|\mathbf{Y}}(C)$). This contribution $\mathbf{X}_{\mathbf{\Gamma}(C)|\mathbf{Y}}(C)$ depends on the biomass concentration C because the absorption coefficient of the suspension determines the fraction of incident light flux that is transmitted along the optical path $\mathbf{\Gamma}(C)|\mathbf{Y}$ (according to Beer law). In the end, the conditional expectation $\mathbf{A_Y}(C) = \mathcal{E}_{\mathbf{X}|\mathbf{Y}}(\mathbf{X}|\mathbf{Y}) = \mathcal{E}_{\mathbf{\Gamma}(C)|\mathbf{Y}}(\mathbf{X}_{\mathbf{\Gamma}(C)|\mathbf{Y}}(C))$ is the rate of photon absorption at location $\mathbf{Y}$, for the biomass concentration C.

Photons absorbed by a micro-algae are converted within the photosynthetic units and the Z-scheme[17,18], leading to the local biomass growth-rate[11,14]

$$\begin{aligned} r_{\mathbf{Y}}(C) &= \mathrm{f}(\mathbf{A_Y}(C), C) \\ &= C\left(\alpha\,\frac{K\,\mathbf{A_Y}(C)}{K+\mathbf{A_Y}(C)} - \beta\,\frac{K_r}{K_r+\mathbf{A_Y}(C)}\right) \\ &= C\left(\alpha\,\frac{K\,\mathcal{E}_{\mathbf{\Gamma}(C)|\mathbf{Y}}(\mathbf{X}_{\mathbf{\Gamma}(C)|\mathbf{Y}}(C))}{K+\mathcal{E}_{\mathbf{\Gamma}(C)|\mathbf{Y}}(\mathbf{X}_{\mathbf{\Gamma}(C)|\mathbf{Y}}(C))} - \beta\,\frac{K_r}{K_r+\mathcal{E}_{\mathbf{\Gamma}(C)|\mathbf{Y}}(\mathbf{X}_{\mathbf{\Gamma}(C)|\mathbf{Y}}(C))}\right) \end{aligned} \tag{9}$$

where α, β, K and K_r are constant parameters that depend on the studied microorganism (see[14] for values in the case of *Chlamydomonas Reinhardtii*).

Due to light absorption and scattering by micro-algae, the field $\mathbf{A_Y}$ is heterogeneous within the volume (less light farther from the fibres and for higher concentrations) and so is the local photosynthetic rate $r_{\mathbf{Y}}(C)$. We address the Monte Carlo computation of $R(C) = \mathcal{E}_{\mathbf{Y}}(r_{\mathbf{Y}}(C))$, the local photosynthetic rate $r_{\mathbf{Y}}(C)$ averaged over locations $\mathbf{Y}$ of the culture volume. The full expression is then

$$R(C) = \mathcal{E}_{\mathbf{Y}} \left(C \left(\alpha \, \frac{K \, \mathcal{E}_{\mathbf{\Gamma}(C)|\mathbf{Y}}(\mathbf{X}_{\mathbf{\Gamma}(C)|\mathbf{Y}}(C))}{K + \mathcal{E}_{\mathbf{\Gamma}(C)|\mathbf{Y}}(\mathbf{X}_{\mathbf{\Gamma}(C)|\mathbf{Y}}(C))} - \beta \, \frac{K_r}{K_r + \mathcal{E}_{\mathbf{\Gamma}(C)|\mathbf{Y}}(\mathbf{X}_{\mathbf{\Gamma}(C)|\mathbf{Y}}(C))} \right) \right) \tag{10}$$

Non-Linear Monte Carlo formulation $R(C)$ is reformulated based on the Taylor expansion of f around x_0. First, independent and equally distributed optical-path random variables $\mathbf{\Gamma}_i(C)|\mathbf{Y}$ are introduced, defining independent and equally distributed contributions $\mathbf{X}_i(C)|\mathbf{Y} = \mathbf{X}_{\mathbf{\Gamma}_i(C)|\mathbf{Y}}(C)$. Then, we expand the non-linear function in Eq. 9 around x_0 chosen as an upper bound of $\mathbf{X}_i(C)|\mathbf{Y}$. Finally, the infinite power series is statistically formulated thanks to the discrete random variable N the order of Taylor expansion:

$$N_{\mathbf{Y}}(C) = B_{\mathbf{X}_1(C)|\mathbf{Y}} + \sum_{i=2}^{+\infty} i \, B_{\mathbf{X}_i(C)|\mathbf{Y}} \prod_{q=1}^{i-1} (1 - B_{\mathbf{X}_q(C)|\mathbf{Y}}) \tag{11}$$

where the $B_{\mathbf{X}_i(C)|\mathbf{Y}}$ are Bernoulli random variables:

$$B_{\mathbf{X}_i(C)|\mathbf{Y}} = \begin{cases} 1 & \text{with probability} \quad P_{\mathbf{X}_i(C)|\mathbf{Y}} = \dfrac{\mathbf{X}_i(C)|\mathbf{Y}}{x_0} \\ 0 & \text{with probability} \quad 1 - P_{\mathbf{X}_i(C)|\mathbf{Y}} \end{cases} \tag{12}$$

In the end, the reformulation is:

$$R(C) = \mathcal{E}_{\mathbf{Y},(\mathbf{\Gamma}_1,\mathbf{\Gamma}_2,\ldots,\mathbf{\Gamma}_{\mathbf{N_Y}})|\mathbf{Y}} \left(w(N_{\mathbf{Y}}) \right) \tag{13}$$

where the Monte Carlo weight function w is

$$w(u) = \begin{cases} C\left[\alpha\,\frac{K\,x_0}{K+x_0} - \beta\,\frac{K_r}{K_r+x_0}\right] & \text{if } u=1 \\ w(1) - \sum_{i=1}^{u-1} C\left[\alpha\,\frac{K^2}{K+x_0}\left(\frac{x_0}{K+x_0}\right)^i + \beta\,\frac{K_r}{K_r+x_0}\left(\frac{x_0}{K+x_0}\right)^i\right] & \text{if } u>1 \end{cases} \tag{14}$$

with $u \in \mathbb{N}$ and α, K, β, K_r, x_0 and C known constants.

Algorithm

Step 1 Uniform sampling of a location $\mathbf{y}$ within the culture volume and index initialisation $n = 1$.

Step 2 Sampling of the n-th optical path contributing to absorption at $\mathbf{y}$: the realisation x_n of $\mathbf{X}_{\mathbf{\Gamma}_n(C)|\mathbf{Y}}(C)$ is computed according to[15].

Step 3 Computation of the probability in Eq. 12, $P = x_n/x_0$, and sampling of a realisation b_n of the Bernoulli random variable $B_{\mathbf{X}_n(C)|\mathbf{Y}}$.

Step 4 If $b_n = 0$, the algorithm loops to step 2 after incrementation of n. Else, the procedure is terminated and the weight $\mathbf{w}(n)$ computed according to Eq. 14.

Simulated configuration Results shown in Extended Data Figure 3 are obtained for kinetics parameters $\alpha = 1.785\,.10^{-9}\ kg/\mu mol_{h\nu}$, $\beta = 4.057\,.10^{-6}\ s^{-1}$, $K = 32000\ \mu mol_{h\nu}/kg/s$ and $K_r = 7500\ \mu mol_{h\nu}/kg/s$. The photobioreactor is a 25L DiCoFluV[13]: reactor diameter $16.5\,cm$, reactor height $1\,m$, optical fibres diameter $1.2\,mm$, fibres height $1\,m$, 979 fibres, hexagonal lattice fibre arrangement with distance $4.8\,mm$ between two fibres axis (see[13,15]), homogeneous surface flux density $25\,\mu mol_{h\nu}/m^2/s$ emitted at fibres surface. The radiative properties (absorption and scattering by micro-algae) are those presented in[10] for *Chlamydomonas Reinhardtii*.

a

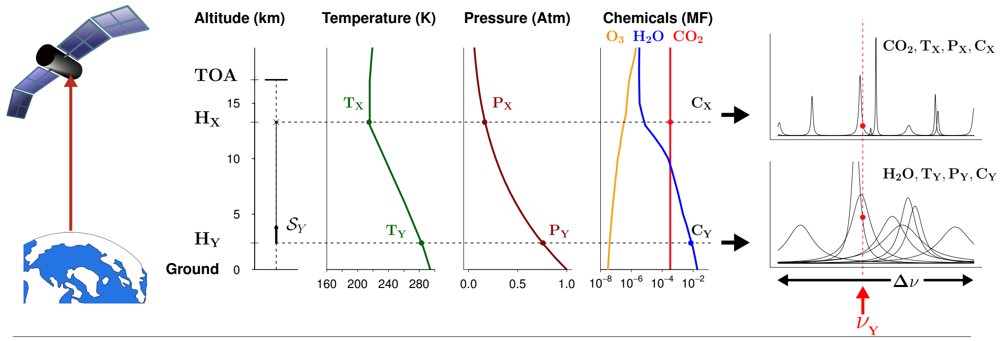

b

Specific intensity at the Top-of-atmosphere (TOA) integrated over a spectral band $\Delta\nu$

$$I(\Delta\nu) = \mathcal{E}_{\mathbf{Y}}\left(\mathcal{S}_Y \, \mathrm{f}\left(\tau_{\mathbf{Y}}\right)\right)$$

$\mathbf{\Gamma_Y}$	**Photon path from altitude $\mathbf{H_Y}$ to TOA**
$\mathcal{S}_{\mathbf{Y}}$	**Radiative source at the start of $\mathbf{\Gamma_Y}$ for one emission-transition $\mathbf{L_Y}(\mathbf{T_Y}, \mathbf{P_Y}, \mathbf{C_Y}, \nu_{\mathrm{Y}})$.**
$\mathrm{f}(\tau_{\mathbf{Y}}) = \exp(-\tau_{\mathbf{Y}})$	**Beer extinction along the path $\mathbf{\Gamma_Y}$**
$\tau_{\mathbf{Y}} = \mathcal{E}_{\mathbf{X}\vert\mathbf{Y}}(\mathbf{X}\vert\mathbf{Y})$	**Optical thickness from altitude $\mathbf{H_Y}$ to TOA at frequency $\nu_{\mathbf{Y}}$ along $\mathbf{\Gamma_Y}$**
$\mathbf{X}\vert\mathbf{Y}$	**Contribution to τ_Y of one absorption-transition $\mathbf{L_X}(\mathbf{T_X}, \mathbf{P_X}, \mathbf{C_X}, \nu_{\mathrm{Y}})$ at altitude $\mathbf{H_X}$**

c

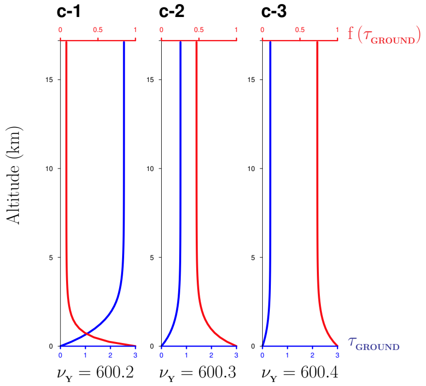

d

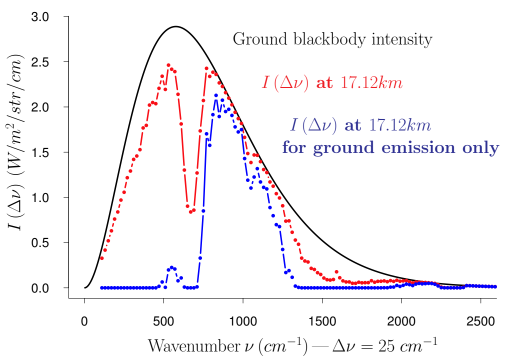

Extended Data Figure 4 | Atmospheric radiative transfert: top-of-atmosphere (TOA) specific intensity (from earth toward the outer space).

a, Measuring and analysing the radiation exiting an atmosphere at its top is archetipal of radiative-transfer physics in both the atmospheric science and astrophysics contexts : reflexion of solar incidence or IR-cooling to space for weather forcasting and climate-change prediction, interpretation of satelite measurements for earth monitoring, inversion of atmospheric profiles for planetary and now exo-planetary studies. For earth at infrared frequencies, photons are emitted by the ground at the surface and by the atmospheric gases at all locations H_Y between surface and top-of-atmosphere. But not all photons emitted upward reach TOA : a fraction is absorbed by the very same gases as those responsible for atmospheric emission, mainly CO_2 and H_2O at all altitudes $H_{X|Y}$ between emission-location H_Y and TOA. The emission and absorption spectra of these molecular gases display very numerous lines that are the result of energy-state transitions : observation bands of spectral width $\Delta\nu$ typically involve several tens to hundred thousands lines of intensities and shapes that strongly depend on altitude via the atmospheric profiles of temperature T, pressure P and gaseous concentrations C.

b, For a given source $\mathcal{S}_Y$ of photons travelling along a path $\mathbf{\Gamma_Y}$ of optical thickness τ_Y, only $\mathcal{S}_Y exp(-\tau_Y)$ is transmitted and $\tau_Y = \mathcal{E}_{X|Y}(X|Y)$ reflects the statistics of all state transitions X (absorption lines) along $\mathbf{\Gamma_Y}$. But these two statistics are combined via the negative exponential function. This is the reason why standard Monte Carlo simulations of atmospheric radiation involve a precomputation phase in which optical thickness is evaluated by adding the contributions of each absorption line in a deterministic manner: the Monte Carlo algorithm itself only deals with the statistics of photon paths. Here, on the contrary, we directly address $\mathcal{S}_Y exp(-\mathcal{E}_{X|Y}(X|Y))$ by non-linearly combining X and Y, i.e. both optical paths and state-transitions in one single algorithm.

c, Dealing with the non-linearity of Beer extinction is the leading question of band-average radiative transfer. There is a double difficulty : i) because of sharp absorption-lines, even in the narrowest bands, the optical thickness varies of orders of magnitude with frequency (compare τ_Y at three frequencies, c-1, c-2, c-3); ii) because of the variations of pressure and composition with altitude, the spatial dependance of τ_Y is difficult to handle, and so is the diversity of its exponential translation : $\mathrm{f}(\tau_Y) \approx 1 - \tau_Y$ in c-3 whereas it is highly non-linear c-1.

d, When applied to a typical state of earth atmosphere, our NLMC algorithm addresses successfully the simulation of band-averaged outgoing IR-radiation (zenith angle, $\Delta\nu = 25cm^{-1}$) without the need of pre-processing the state-transition database (here Hitran) : for each sampled path, several state-transitions are sampled in the database, depending on the sampled order of the Taylor expansion of the exponential (using the null-collision approach). In red, the band-averaged specific intensity for several bands covering the whole infrared ; in blue, the part of this intensity due to photons emitted at the surface (close to red at frequencies where the atmosphere is nearly transparent).

SI4 — Atmospheric radiative transfer: top-of-atmosphere specific intensity (from earth toward the outer space)

This example is fully detailed in Galtier *et al.* 2015[19].

The problem (see Extended Data Figure 4). Photons are emitted either by a surface (here the ground) or by the volume (the atmosphere). The radiation $I(\Delta\nu)$ perceived at the observation location TOA is the fraction of all these photons that have a frequency inside the observation-band $\Delta\nu$ and reach the sensor, i.e. which are not absorbed by the atmosphere in between. When no scattering occurs (clear sky), photon-paths are straight lines and the fraction of the photons of frequency ν_y that travel (let say along the strict vertical) from altitude H_y to TOA is

$$exp(-\tau_y)$$

where

$$\tau_y = \int_{H_y}^{TOA} dx \sum_{j_x=1}^{N_t} h_{\nu_y,j_x}(x)$$

This exponential extinction is that of Beer law where τ_y is the monochromatic optical thickness, i.e. the sum of all transition cross-sections $h_{\nu_y,j_x}(x)$ at all intermediate altitudes x. In this example, the random variable $\mathbf{Y}$ is a vector that gathers the altitude $H_\mathbf{Y}$ of emission, the photon-path $\mathbf{\Gamma_Y}$ from $H_\mathbf{Y}$ to TOA and frequency $\nu_\mathbf{Y}$ of emission. Similarly, $\mathbf{X}$ gathers all the description of an absorption event, altitude $H_\mathbf{X}$ and index of absorption-transition $j_\mathbf{X}$.

In standard Monte Carlo approaches, τ_y is either precomputed and tabulated, or is easily computable from tabulated values of the absorption coefficient ($k = \sum_{j_x=1}^{N_t} h_{\nu_y,j_x}$). Then the Monte Carlo algorithm deals only with the sampling of $\mathbf{Y}$ and the weight function has the form

$$w(y) = \mathcal{S}_y exp(-\tau_y)$$

$\mathcal{S}_\mathbf{Y}$ is the source associated with the emission $\mathbf{Y}$ (Planck's function at the local temperature times

an emissive power that depends on concentration, pressure and temperature). The sum over the N_t transitions j_x is thus not handled by standard Monte Carlo algorithms, despite the fact that this sum has all the features inviting to make use of statistical approaches: N_t is huge, typically of the order of 10^6, and the deterministic pre-calculations can be computationally very demanding (and are to be re-performed when testing each new spectroscopic assumption). No attempt has been made so far to address this sum statistically, together with the photon-path statistics because these two statistics are combined via the non-linearity of the exponential extinction.

Non-Linear Monte Carlo formulation So the present question is to design a Monte Carlo algorithm performing the summation $\sum_{j=1}^{N_t} h_{\nu,j}$ together with the integrals over frequency, emission location and absorption location, despite of the nonlinearity of Beer extinction. This starts by looking at τ_y in statistical terms : the integral over altitudes $H_{\mathbf{X}}$ is transformed into the sampling of absorption locations, and the sum $\sum_{j_x=1}^{N_t} h_{\nu_y,j_x}$ into the sampling of absorption-transitions. This double sampling is formally summarised into the random variable $\mathbf{X}$. To get a sample x of $\mathbf{X}$, one first samples $H_{\mathbf{X}}$ and $j_{\mathbf{X}}$. Then x is the value that the optical thickness τ_y would have if the atmosphere was homogeneous at the thermodynamic conditions of altitude H_x and if all transitions were identical to j_x. This random variable depends on $\mathbf{Y}$ and its expectation, knowing $\mathbf{Y} = y$, is $\mathcal{E}(\mathbf{X}|\mathbf{Y} = y) = \tau_y$. We therefore get $I\,(\Delta\nu) = \mathcal{E}_{\mathbf{Y}}\,(\mathcal{S}_{\mathbf{Y}}\;exp(\,-\,\mathcal{E}_{\mathbf{X}|\mathbf{Y}}(\mathbf{X}|\mathbf{Y})\,))$

Algorithm A strict application of the solution described in *Methods* implies to first sample $\mathbf{Y}$ (as in a standard Monte Carlo algorithm), then sample the degree n of one monomial in the Taylor expansion of the negative-exponential function f, and draw n independent samples $x_1, x_2 ... x_n$ of $\mathbf{X}$, i.e n absorption locations H_{x_q} (between emission location and TOA) and n transition indexes j_{x_q}:

Step 1 sample a frequency,

Step 2 sample an emission-altitude,

Step 3 sample an order n of the development of the exponential,

Step 4 sample n paired absorption-altitudes, transitions.

Null-collision reformulation Instead of implementing this solution in this straightforward manner, we chose to use null-collisions[20]. The same quantities are sampled, but the order is different, leading to quite intuitive physical pictures. The first step is still to sample a frequency, but then we implement a backward tracking algorithm, starting from the observation altitude. We sample a first collision-altitude as if photons were emitted at the observation location in the downward direction in an homogeneous atmosphere. Then we sample a transition and a statistical test is made to determine whether the algorithm stops at this altitude or continues (a Bernoulli test to determine whether the collision is a true one or a null-collision). If it continues, this first collision-altitude and first transition have the status of one of the n absorption-altitudes and transitions in the above presented algorithm. Then a next collision-location is sampled, together with a next transition, etc. When the algorithm stops after n collisions, the final altitude is interpreted as the emission-altitude. So n and the emission-altitude are not sampled first: they are sampled by the successive Bernoulli tests as in a standard backward-tracking multiple-scattering algorithm.

The final algorithm

Step 1 Initialisation of the current altitude at $H_x \leftarrow TOA$.

Step 2 Uniform sampling of a frequency ν_y in the considered infrared band (or the whole infrared).

Step 3 Exponential sampling of a travelled distance d before absorption for a photon of frequency ν_y travelling from H_x in the backward direction within a virtual homogeneous atmosphere of absorption coefficient $\hat{k}_{\nu_y}$.

Step 4 If the travelled distance leads the photon to hit the surface, $H_x \leftarrow 0$ and the algorithm jumps to *Step 7*. Otherwise $H_x \leftarrow H_x - d$.

Step 5 Sampling of a state-transition j_x according to $\mathcal{P}(1), \mathcal{P}(2)...\mathcal{P}(N_t)$ (see[19]).

Step 6 Bernoulli trial of probability $P_a = \frac{h_{\nu_y, j_x}}{\hat{k}_{\nu_y}\mathcal{P}(j_x)}$ to decide whether the algorithm jumps to *Step 7* or *Step 3*.

Step 7 $H_y \leftarrow H_x$ and the algorithm stops with $\hat{w} = \Delta\nu B_y$ where B_y is the Planck function at frequency ν_y for the atmospheric temperature at H_y.

Simulated configuration The simulation results in Extended Data Fig. 4 have been obtained using the HITRAN spectroscopic database. All other parameters are given in Galtier *et al.* 2015[19].

Computational performance As far as computational costs are concerned, our computations did not require to first scan HITRAN and pre-compute absorption coefficients at all altitudes and all frequencies before running the Monte Carlo code for transfer since our Monte Carlo code handles both simultaneously despite the nonlinearity of the exponential extinction. The computational benefit is very significant when studying the effects of new spectroscopic data or new line-shape assumptions. Otherwise, for earth applications and fixed spectroscopic assumptions, there is no problem associated to the pre-computation of absorption coefficients and to their tabulation as function of molecular composition and thermodynamic state. But for combustion or astrophysics applications, thinking in particular of exoplanets, the diversity of compositions is extremely wide, temperatures can by high, imply the use of much larger spectroscopic databases, including hot lines, and pre-computation/tabulation of absorption coefficients is a today's challenge in itself. Our nonlinear Monte Carlo suppresses this need[19].

SI5 — Gas kinetics

The problem We consider a gas of interacting particles, with collisions following Maxwell model, and a cross section $\sigma(\vec{c},\vec{c^*}) = \frac{\kappa}{4\pi\|\vec{c}-\vec{c^*}\|}$. Particles are confined within an harmonic static trap of pulsation ω so that acceleration at position $\vec{r}$ is spring-like: $\vec{a}(\vec{r}) = -\omega^2\vec{r}$.

We follow the distribution function $f(\vec{r},\vec{c},t)$ at location $\vec{r}$, velocity $\vec{c}$ at time t. We consider it known at some time t_I, either constrained at local equilibrium, according to:

$$f_{\mathbf{LEQ}}/n_I = p_{\mathcal{N}(\vec{u}_I,c_{q,I}^2)} \tag{15}$$

or, out of equilibrium, according to:

$$f_{\mathbf{BKW}}/n_I = \frac{1}{3}\frac{(\vec{c}-\vec{u}_I)^2}{\frac{c_{q,I}^2}{5/3}}\, p_{\mathcal{N}\left(\vec{u}_I,\frac{c_{q,I}^2}{5/3}\right)} \tag{16}$$

where n_I denotes density, $\vec{u}_I$ denotes the mean velocity and $c_{q,I}$ denotes the mean square speed at $\vec{r}$ at time t_I ; $p_{\mathcal{N}(\mu,\sigma^2)}$ denotes probability density function of a Gaussian random variable, with expectation μ and variance σ^2.

For the case in fig. 3a, we set $\omega = 0$, and n_I, $\vec{u}_I$ and $c_{q,I}$ are set homogeneous, corresponding to the case studied par Krook and Wu[21,22]. Starting from $f_{\mathbf{BKW}}$, the gas relaxes to equilibrium $f_{\mathbf{LEQ}}$.

Adimensional time is $\kappa n_I t$ and adimensional distribution function is $\frac{1}{n_I}\left(\sqrt{2\pi}c_{q,I}\right)^3 f$.

For the case in fig. 3b, we set $\omega = 2\pi$, and n_I, $\vec{u}_I$ and $c_{q,I}$ are set heterogeneous to correspond to one state of the undamped oscillation (*breathing mode*[23]):

$$\begin{aligned} n_I &= p_{\mathcal{N}(\vec{0},\frac{c^2_{q,EQ}}{\omega^2})} \\ \vec{u}_I &= \epsilon\omega\vec{r} \\ c_{q,I} &= \sqrt{1-\epsilon^2}\, c_{q,EQ} \end{aligned} \tag{17}$$

with $\epsilon = \frac{\Delta c_q^2}{c^2_{q,EQ}}$ where Δc_q^2 is the maximal deviation of c_q^2 from its equilibrium value $c^2_{q,EQ}$.

With these initial values, and starting from local equilibrium $f_{\mathbf{LEQ}}$, the local equilibrium remains unbroken and the gas displays the undamped oscillation for any value of the cross section. Conversely, starting from out of equilibrium $f_{\mathbf{BKW}}$, any positive value of cross section will lead to dampened oscillations.

Adimensional time is $\frac{\omega}{2\pi}t$, adimensional distribution function is $\left(2\pi\frac{c^2_{q,EQ}}{\omega}\right)^3 f$ and adimensional parameter for cross-section is $\frac{\omega^2}{c^3_{q,EQ}}\kappa$.

Simulation results are given for $\epsilon = 0.2$ and $\frac{\omega^2}{c^3_{q,EQ}}\kappa = 3$.

Non-Linear Monte Carlo formulation The distribution function obeys the Boltzmann equation:

$$\begin{aligned} &\frac{\partial f}{\partial t} + \vec{c}\cdot \vec{\text{grad}}_{\mathcal{R}}(f) + \text{div}_{\mathcal{C}}(f\vec{a}) \\ &\quad = \int_{\mathcal{C}} d\vec{c}_* \int_{4\pi} d\vec{u}\ \|\vec{c}-\vec{c}_*\|\ \sigma(\vec{c},\vec{c}_*)\left(-ff^* + f^\alpha f^\beta\right) \end{aligned} \tag{18}$$

where $f \equiv f(\vec{r},\vec{c},t)$, $f^* \equiv f(\vec{r},\vec{c}_*,t)$, $f^\alpha \equiv f(\vec{r},\vec{c}_\alpha,t)$ and $f^\beta \equiv f(\vec{r},\vec{c}_\beta,t)$.

Velocities $\vec{c}_\alpha$ and $\vec{c}_\beta$ are functions of $\vec{c}$, $\vec{c}_*$ and $\vec{u}$: $\vec{c}_\alpha = \frac{1}{2}(\vec{c} + \vec{c}_* + \|\vec{c} - \vec{c}_*\|\, \vec{u})$, $\vec{c}_\beta = \frac{1}{2}(\vec{c} + \vec{c}_* - \|\vec{c} - \vec{c}_*\|\, \vec{u})$.

The usual PDE expression of this model, given above, can be translated into its Fredholm counterpart, following:

$$
\begin{aligned}
f(\vec{r}, \vec{c}, t) = & \int_{-\infty}^{t} dt'\, \hat{\nu}(t')\, \exp\left(- \int_{t'}^{t} dt''\, \hat{\nu}(t'') \right) \times \\
& \Bigg[\mathcal{H}(t_I - t')\, f(\vec{r}_b(t_I), \vec{c}_b(t_I), t_I) \\
& + \mathcal{H}(t' - t_I) \left(1 - \frac{\nu(t')}{\hat{\nu}(t')} \right) f\left(\vec{r}_b(t'), \vec{c}_b(t'), t'\right) + \frac{s(t')}{\hat{\nu}(t')} \Bigg]
\end{aligned} \tag{19}
$$

with

$$
\begin{aligned}
\nu(t') &= \int_{\mathcal{C}} d\vec{c}_* \int_{4\pi} d\vec{u}\, \|\vec{c}_b(t') - \vec{c}_*\|\, \sigma(\vec{c}_b(t'), \vec{c}_*) f(\vec{r}_b(t'), \vec{c}_*, t') \\
s(t') &= \int_{\mathcal{C}} d\vec{c}_* \int_{4\pi} d\vec{u}\, \|\vec{c}_b(t') - \vec{c}_*\|\, \sigma(\vec{c}_b(t'), \vec{c}_*) f(\vec{r}_b(t'), \vec{c}_\alpha(t'), t') f(\vec{r}_b(t'), \vec{c}_\beta(t'), t')
\end{aligned} \tag{20}
$$

where $\vec{r}_b$ and $\vec{c}_b$ are location and velocity corresponding to the ballistic path through $\vec{r}$ at time t with velocity $\vec{c}$, such as:

$$
\begin{cases}
\partial_{t'} \vec{r}_b(t') = \vec{c}_b(t') \\
\partial_{t'} \vec{c}_b(t') = \vec{a}(\vec{r}_b(t'))
\end{cases}
$$

and $\vec{r}_b(t) = \vec{r}$, $\vec{c}_b(t) = \vec{c}$.

The product $f(\vec{r}_b(t'), \vec{c}_\alpha(t'), t') f(\vec{r}_b(t'), \vec{c}_\beta(t'), t')$ has been treated following the NLMC expansion exposed in Methods.

The exponential term is handled using null collisions technique, as exposed in SI4. The upper bound for cross section is set at the value it takes at the center of the gas cloud at time of maximal contraction of the undamped oscillation :

$$\hat{\nu} = \frac{\omega^3}{\left(2\pi c_{q,EQ}^2(1-\epsilon)\right)^{3/2}}\,\kappa$$

Algorithm According to eq. 19, three random variables are defined for the Monte Carlo estimates: T' for t', $\vec{U}$ for $\vec{u}$ and $\vec{C}_*$ for $\vec{c}_*$, with:

$$\begin{array}{lcll} p_{T'}(t') & = & \hat{\nu}\ \exp\left(-\hat{\nu}(t-t')\right) & \text{over }]-\infty,t] \\ p_{\vec{U}}(\vec{u}) & = & \frac{1}{4\pi} & \text{over the unit sphere} \\ p_{\vec{C}^*}(\vec{c}_*) & = & p_{\mathcal{N}(\vec{u}_{Boltz},c^2_{q,Boltz})} & \text{over velocity space} \end{array} \qquad (21)$$

where $\vec{u}_{Boltz}$ et $c_{q,Boltz}$ are respectively the mean velocity and the mean square speed at the considered time, and set to the values predicted in the case of undamped oscillation.

$f(\vec{r}, \vec{c}, t)$ can be estimated by this recursive algorithm:

Initialisation Sample a date t'.

Recursion termination: If $t' \leqslant t_I$ return $f(\vec{x}_I, \vec{c}_I, t_I)$.

Recursion

Sample a velocity $\vec{c}_*$ and a unit vector $\vec{u}$.

Compute the ballistic solution $\vec{x}_b(t')$ and $\vec{c}_b(t')$.

Estimate $\mathsf{f}_* \leftarrow f(\vec{x}_b(t'), \vec{c}_*, t')$.

Set

$$\begin{aligned} \mathsf{n} &\leftarrow \left(\|\vec{c}_b(t') - \vec{c}_*\| \; \sigma(\vec{c}_b(t'), \vec{c}_*, \vec{u}) \; \mathsf{f}_*\right) \Big/ \left(p_{\vec{C}_*}(\vec{c}_*) p_{\vec{U}}(\vec{u})\right) \\ \mathsf{Q} &\leftarrow \mathsf{n}/\hat{\nu} \end{aligned} \tag{22}$$

If $\mathsf{Q} \in [0, 1]$, then set $\mathsf{P} \leftarrow \mathsf{Q}$ else set $\mathsf{P} \leftarrow \frac{\mathsf{Q}}{2\mathsf{Q}-1}$.

Sample a standard uniform r.

If $r > \mathsf{P}$, then estimate $\mathsf{f_b} \leftarrow f(\vec{x}_b(t'), \vec{c}_b(t'), t')$ and return $\frac{1-\mathsf{Q}}{1-\mathsf{P}} \; \mathsf{f_b}$,

Else set

$$\begin{aligned} \vec{c}_\alpha(t') &\leftarrow \tfrac{1}{2}(\vec{c}_b(t') + \vec{c}_* + \|\vec{c}_b(t') - \vec{c}_*\| \, \vec{u}) \\ \vec{c}_\beta(t') &\leftarrow \tfrac{1}{2}(c_b\vec{(t')} + \vec{c}_* - \|\vec{c}_b(t') - \vec{c}_*\| \, \vec{u}) \end{aligned} \tag{23}$$

Estimate $\mathsf{f}_\alpha \leftarrow f(\vec{x}_b(t'), \vec{c}_\alpha(t'), t')$.

Estimate $\mathsf{f}_\beta \leftarrow f(\vec{x}_b(t'), \vec{c}_\beta(t'), t')$.

Return $(\mathsf{Q}/\mathsf{P}) \, (\mathsf{f}_\alpha \, \mathsf{f}_\beta / \mathsf{f}_*)$

1. Steinfeld, A. Solar thermochemical production of hydrogen–a review *Sol. Energy*. **78**, 603 (2005)

2. Müller, R. et al. Transient heat transfer in a directly-irradiated solar chemical reactor for the thermal dissociation of ZnO. *Appl. Therm. Eng.* **28**, 524 (2008)

3. Schunk, L.O. et al. Heat transfer model of a solar receiver-reactor for the thermal dissociation of ZnO-Experimental validation at 10 kW and scale-up to 1 MW. *Chem. Eng. J.* **150** 502 (2009)

4. Pitz-Paal, R. et al. Heliostat field layout optimization for high-temperature solar thermochemical processing. *Sol. Energy*. **85**, 334 (2011)

5. Delatorre, J. et al. Monte Carlo advances and concentrated solar applications. *Sol. Energy*. **103**, 653 (2014)

6. Farges, O. et al. Life-time integration using Monte Carlo Methods when optimizing the design of concentrated solar power plants. Sol. Energy 113, 57–62 (2015).

7. Charon, J. et al. Monte Carlo implementation of Schiff's approximation for estimating radiative properties of homogeneous, simple-shaped and optically soft particles: Application to photosynthetic micro-organisms. J. Quant. Spectrosc. Radiat. Transf. 172, 3–23 (2016).

8. Schiff, L. I. Approximation Method for High-Energy Potential Scattering. *Physical Review*. (1956)

9. van de Hulst, H. C. Light scattering by small particles. *Dover Publication, Inc.*. (1981)

10. Dauchet, J. and al. Calculation of the radiative properties of photosynthetic microorganisms. *J. Quant. Spectrosc. Radiat. Transfer*. (2015)

11. Dauchet, J., Cornet, J.-F., Gros, F., Roudet, M. and Dussap, C.-G. Chapter One – Photobioreactor Modeling and Radiative Transfer Analysis for Engineering Purposes. Adv. Chem. Eng. 48, 1–106 (2016).

12. Bohren, C. F. and Huffman, D. R. Absorption and scattering of light by small particles. *New York, Wiley-Interscience*. (1983)

13. Cornet J.F. Calculation of optimal design and ideal productivities of volumetrically lightened photobioreactors using the constructal approach. *Chem. Eng. Sci.* **65** 985 (2010)

14. Takache, H. et al. Kinetic modeling of the photosynthetic growth of Chlamydomonas reinhardtii in a photobioreactor. *Biotechnol. Prog.* **28** 681 (2012)

15. Dauchet, J. et al. The practice of recent radiative transfer Monte Carlo advances and its contribution to the field of microorganisms cultivation in photobioreactors. *J. Quant. Spectrosc. Radiat. Transfer*. **128** 52 (2013)

16. Delatorre, J. et al. Monte Carlo advances and concentrated solar applications. *Sol. Energy*. **103**, 653 (2014)

17. Hatch, M.D. et al. Photosynthesis and photo-respiration. *Wiley-Interscience* (1971)

18. Cornet J.F. & Dussap C.G. A Simple and reliable formula for assessment of maximum volumetric productivities in photobioreactors *Biotechnol. Prog.* **25** 424 (2009)

19. Galtier, M. et al. Radiative transfer and spectroscopic databases: a line-sampling Monte Carlo approach. J. Quant. Spectrosc. Radiat. Transf. (2015).

20. Galtier, M. et al. Integral formulation of null-collision Monte Carlo algorithms. J. Quant. Spectrosc. Radiat. Transf. 125, 57–68 (2013).

21. Krook, M. & Wu, T. T. Formation of Maxwellian Tails. Phys. Rev. Lett. 36, 1107–1109 (1976).

22. Krook, M. & Wu, T. T. Exact solutions of the Boltzmann equation. Phys. Fluids 20, 1589–1595 (1977).

23. Guéry-Odelin, D., Muga, J. G., Ruiz-Montero, M. J. & Trizac, E. Nonequilibrium Solutions of the Boltzmann Equation under the Action of an External Force. Phys. Rev. Lett. 112, 180602 (2014).
